# Supplementary material for: An innovative engineered IL-10 monomer strengthens T cell-mediated anti-tumor responses through anti-PD-1 cis-delivery
Source: Cell Rep Med. 2025 Dec 16;7(1):102515. doi: 10.1016/j.xcrm.2025.102515 (PMC12866158; doi:10.1016/j.xcrm.2025.102515)
Supplement: Document S1. Figures S1–S7 [file mmc1.pdf]

Cell Reports Medicine, Volume *7*

Supplemental information

An innovative engineered IL-10 monomer strengthens T cell-mediated anti-tumor responses through anti-PD-1 *cis*-delivery

Ce Gu, Jian Guo, Chang Zhou, Peipei Hu, Xiaodong Wu, Jiaojiao Ding, Xinxin Zhou, Liao Zeng, Wen Yu, Yingye Ou, Linhui Ye, Mengying Liang, Yue Huang, Jiatian Li, Zhe Zhang, Wentao Deng, Baiguang Ren, Yingpei Zhang, Li Wang, Xuejiao Chen, Yingxing Duan, Zhe Han, Yang Leng, Hongxin Li, Kongzhen Hu, Yongting Huo, and Di Lu

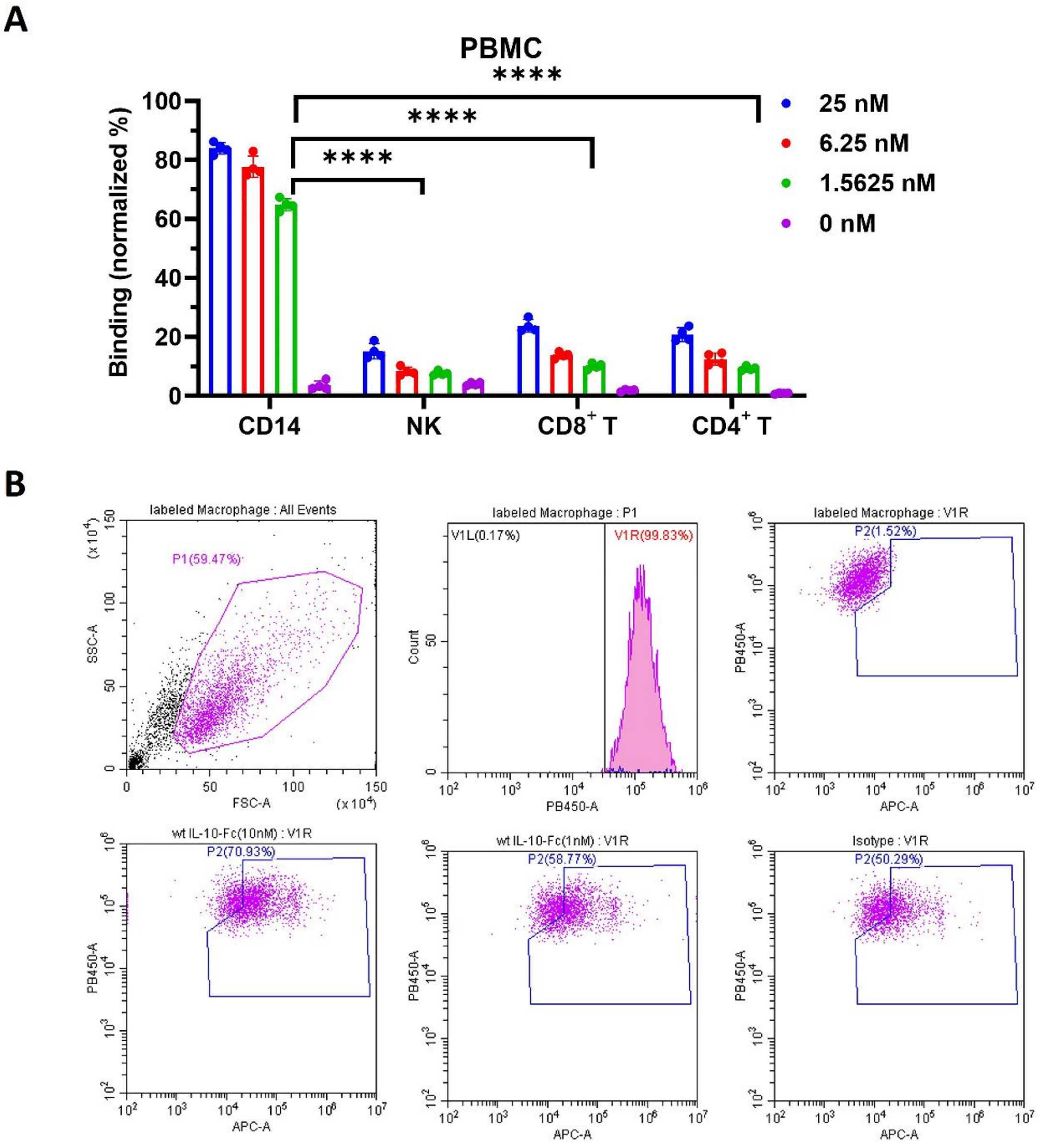

Figure S1. PBMC binding and representative gating strategies for macrophage-mediated RBC uptake. Related to Figure 1.

(A) PBMC cells from another donor were incubated with wt IL-10-Fc in vitro. Protein binding to $CD4^+$ T, $CD8^+$ T, NK or CD14 cells was detected by flow cytometric analysis. (B) Gating was performed on the dual-channel scatter plot. Cells that are $PB450^+$ APC^+ within the P2 gate are macrophages that have phagocytosed red blood cells, and the experiment conducts quantitative and statistical analysis based on the proportion of this cell population. (PB450 is the fluorescence channel corresponding to the macrophage-labeling dye, and APC is the fluorescence channel corresponding to the red blood cell-labeling dye.) Data are reported as the mean ± SEM. Statistical analyses performed by one-way ANOVA with Dunnett's multiple comparisons tests (****$p \leq 0.0001$).

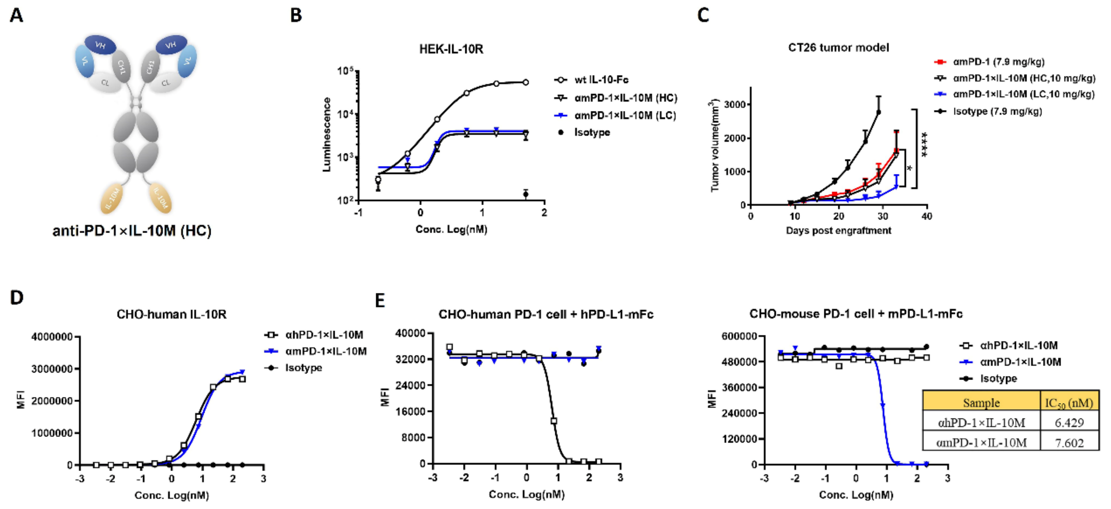

Figure S2. Anti-mPD-1×IL-10M (LC) displayed superior anti-tumor efficacy and exhibited comparable biological activities to anti-hPD-1×IL-10M (LC). Related to Figure 3.

(A) Schematic diagram of anti-PD-1×IL-10M (HC). IL-10M is linked to the C-terminus of the anti-PD-1 antibody heavy chain via (G4S)4G. (B) The bioactivity of the indicated proteins was detected using HEK-IL-10R reporter cells. (C) Balb/c mice were inoculated with 1×10^5 CT26 tumor cells. Tumor-bearing mice (n = 8/group) were intraperitoneally treated with indicated proteins on days 9, 12 and 15. The tumor volume of mice was measured as indicated. (D) Binding activity of indicated proteins to CHO-human IL-10R cells was analyzed by flow cytometric analysis. (E) Blocking activity of indicated proteins on PD-L1 protein binding to CHO-human PD-1 cells (left) or CHO-mouse PD-1 cells (right) was analyzed by flow cytometric analysis. Results are shown as mean ± SEM. Statistical analyses performed by two-way ANOVA with Dunnett's multiple comparisons tests (*$p \leq 0.05$, ****$p \leq 0.0001$).

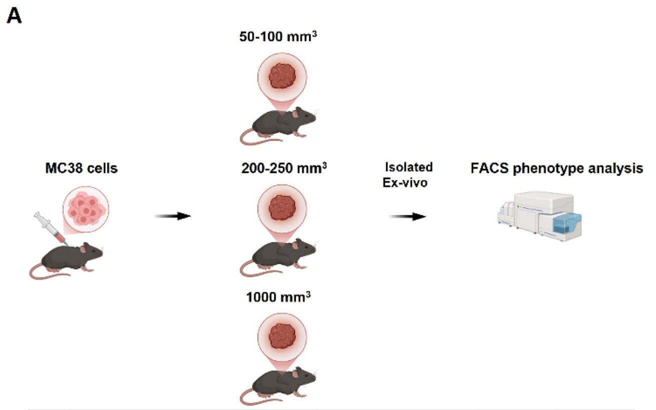

Tumor volume	CD45⁺ %	CD8⁺ %	PD-1⁺ %	IL-10R⁺ %	TIM-3⁺ %	TOX⁺ %
50-100 mm³	71.83	4.11	52.15	21.63	17.62	34.81
200-250 mm³	54.52	3.17	64.21	25.04	14.73	39.76
1000 mm³	46.79	6.04	82.1	46.53	23.86	91.43

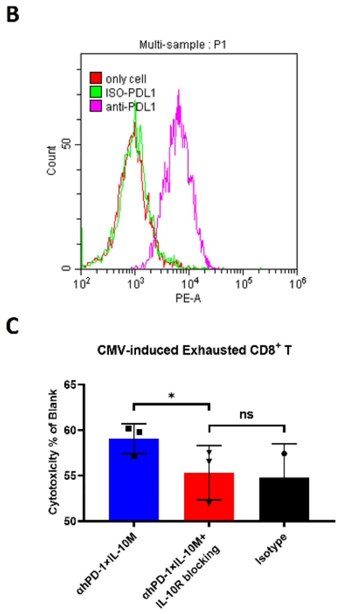

Figure S3. Schematic diagram of isolating tumor infiltrating lymphocytes from MC38 tumors and the expression of PD-L1 and cytotoxicity in PANC.05.04 cells. Related to Figure 4.

(A) C57BL/6 mice were inoculated with 3×10^5 MC38 tumor cells. Tumor infiltrating lymphocytes (TILs) were isolated from MC38 tumor tissues with a volume of 50-100 mm³, 200-250 mm³ and 1000 mm³ using density gradient centrifugation. The proportion of TILs was detected by flow cytometric analysis. Then isolated the CD8⁺ TILs using mouse CD8 Microbeads (Milton Biotec). The proportion of CD8⁺, PD-1⁺ CD8⁺, IL-10R⁺ CD8⁺, TIM-3⁺ CD8⁺ and TOX⁺ CD8⁺ cells were detected by flow cytometric phenotypic analysis. (B) The expression of PD-L1 in PANC.05.04 cells by flow cytometry. (C) CMV-induced exhausted CD8⁺ T were co-cultured with PANC pre-loaded with pp65. Flow cytometric analysis was used to detect the killing efficiency of PANC.05.04 tumor cells under the action of the candidate molecule alone and the candidate molecule combined with the IL-10R inhibitor. Results are reported as the mean ± SEM. Statistical analyses performed by paired Student's t test. (ns, not significant, $^*p \leq 0.05$).

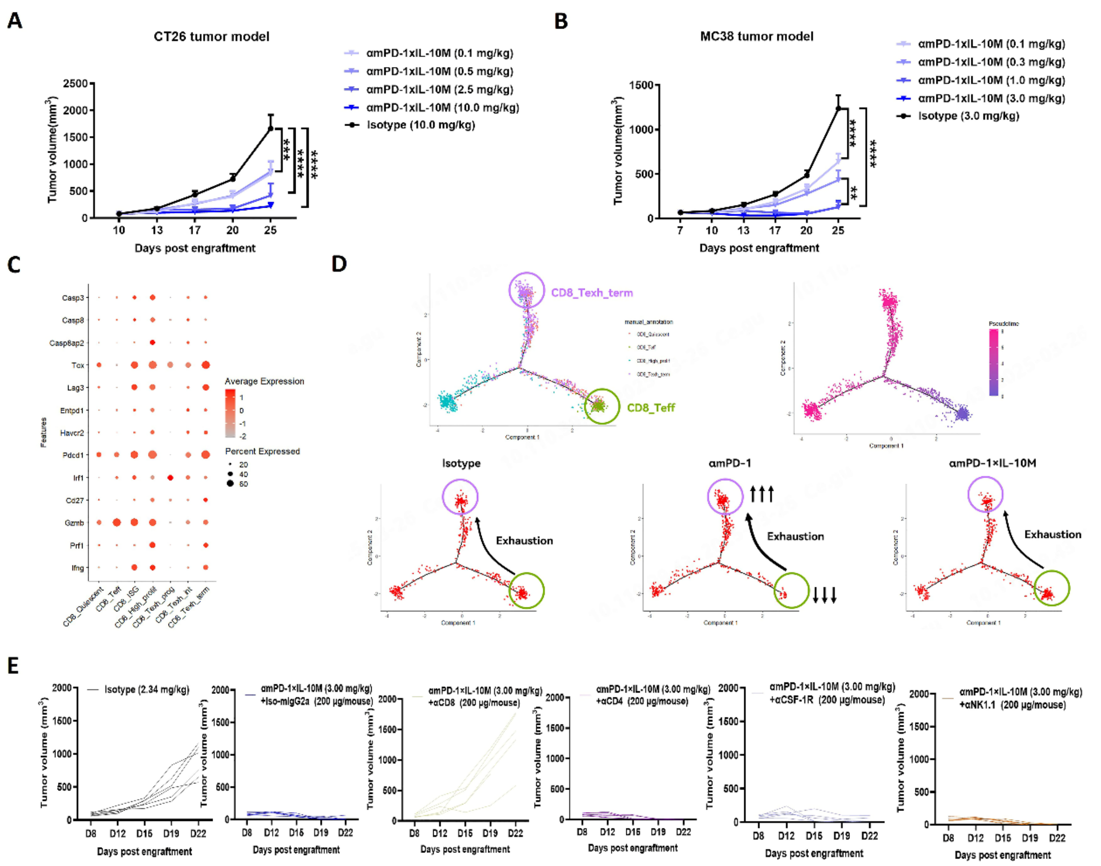

Figure S4. Anti-PD-1×IL-10M exerts anti-tumor efficacy depends on intratumoral CD8$^+$ T cells. Related to Figure 5.

(A) Balb/c mice were inoculated with 1×10^5 CT26 tumor cells. Tumor-bearing mice (n = 9/group) were intraperitoneally treated with different doses of anti-mPD-1×IL-10M proteins on days 10, 13 and 17. The tumor volume of mice was measured as indicated. (B) C57BL/6 mice were inoculated with 3×10^5 MC38 tumor cells. Tumor-bearing mice (n = 9/group) were intraperitoneally treated with different doses of anti-mPD-1×IL-10M proteins on days 7, 10 and 13. The tumor volume of mice was measured as indicated. (C-D) Single-cell sequencing analysis of immune cells in MC38 tumors. (C) According to the expression of corresponding markers, CD8$^+$ T cells were classified into seven types of T cells with different states and functions. CD8_Quiescent, resting CD8$^+$ T cells. CD8_Teff, effector CD8$^+$ T cells. CD8_ISG, expressing interferon responsive genes CD8$^+$ T cells. CD8_High-prolif, high proliferative capacity CD8$^+$ T cells. CD8_Texh-prog, precursor exhausted CD8$^+$ T cells. CD8_Texh-int, intermediate exhausted CD8$^+$ T cells. CD8_Texh-term, terminally exhausted CD8$^+$ T cells. (D) Pseudo temporal analysis of the indicated proteins. (E) Individual tumor growth curves of Fig. 5i. Results are shown as mean ± SEM. Statistical analyses performed by two-way ANOVA with Dunnett's multiple comparisons tests (**$p \leq 0.01$, ***$p \leq 0.001$, ****$p \leq 0.0001$).

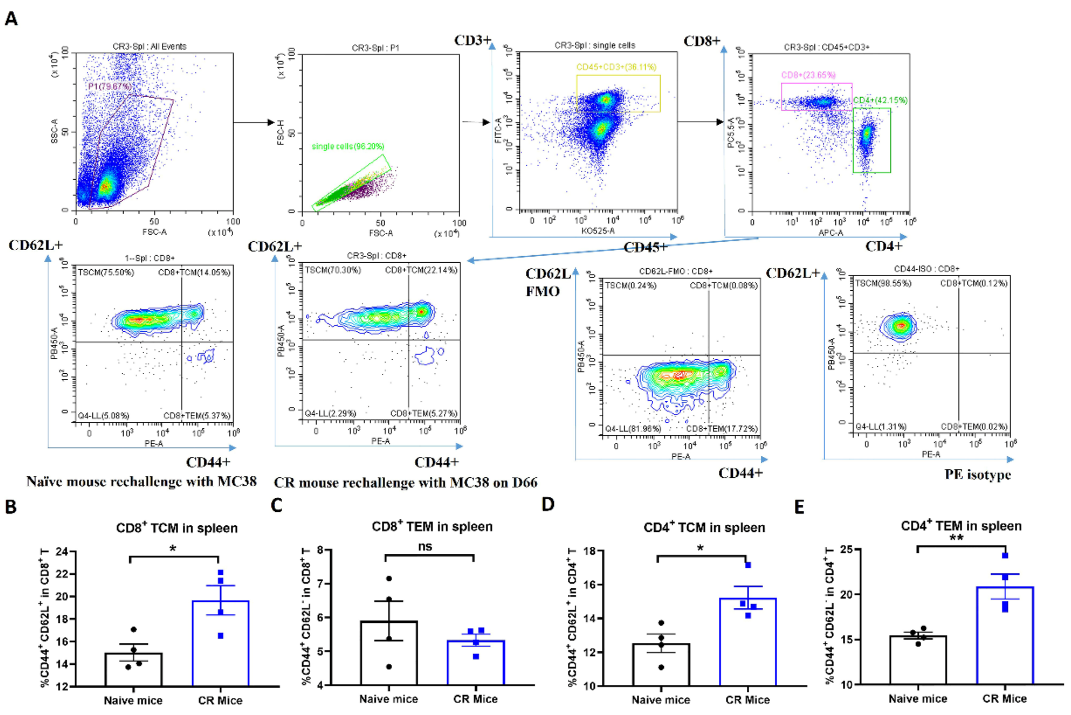

Figure S5. Representative gating strategies and flow cytometry analysis for immunophenotyping of murine spleen samples. Related to Figure 6.

Splenocytes were isolated from treated mice and the immunophenotyping of cell populations was conducted. (A) Gating of $CD8^+$ T and $CD4^+$ T cell populations, namely central memory $CD8^+$ T cells ($CD8^+$ TCM; $CD3^+/CD8^+/CD44^+/CD62L^+$), central memory $CD4^+$ T cells ($CD4^+$ TCM; $CD3^+/CD4^+/CD44^+/CD62L^+$), effector memory $CD8^+$ T cells ($CD8^+$ TEM; $CD3^+/CD8^+/CD44^+/CD62L^-$) and effector memory $CD4^+$ T cells ($CD4^+$ TEM; $CD3^+/CD4^+/CD44^+/CD62L^-$). (B-E) After rechallenging the cured mice with MC38 tumor cells, the changes in the proportion of memory T cells in the mice's spleens were systematically analyzed using flow cytometry. Results are shown as mean ± SEM. Statistical analyses performed by one-way ANOVA with Dunnett's multiple comparisons tests (ns, not significant; $*p \leq 0.05$; $**p \leq 0.01$). CR (Complete Response); Spl (Splenocytes).

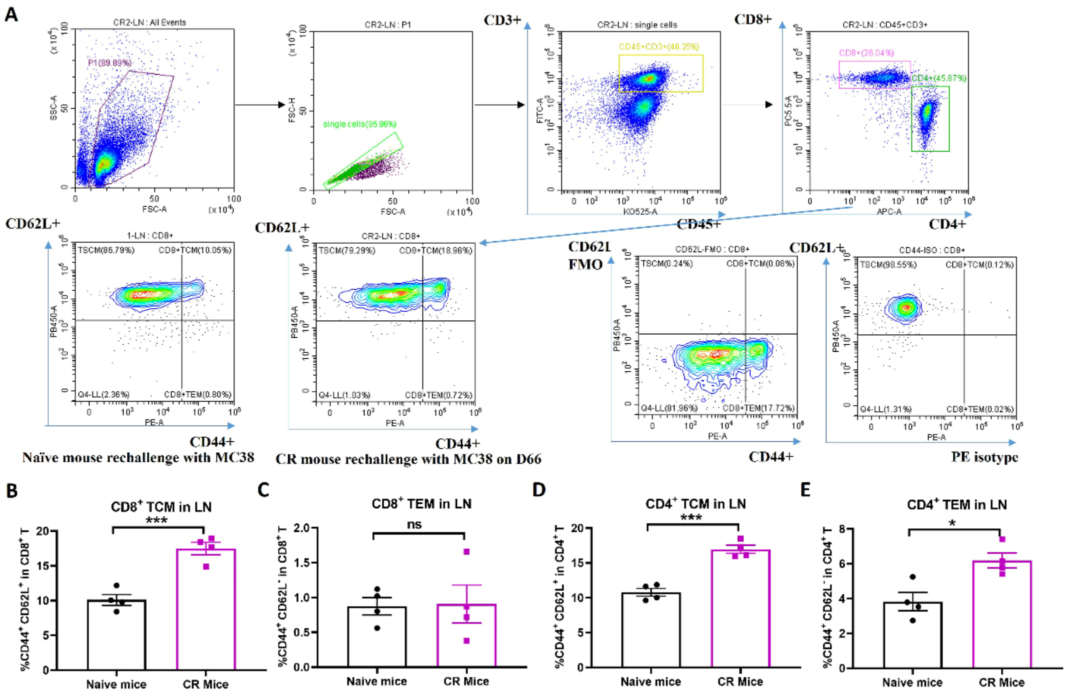

Figure S6. Representative gating strategies and flow cytometry analysis for immunophenotyping of murine lymph node samples. Related to Figure 6.

Lymph node cells were isolated from treated mice and the immunophenotyping of cell populations was conducted. (A) Gating of $CD8^+$ T and $CD4^+$ T cell populations, namely central memory $CD8^+$ T cells ($CD8^+$ TCM; $CD3^+/CD8^+/CD44^+/CD62L^+$), central memory $CD4^+$ T cells ($CD4^+$ TCM; $CD3^+/CD4^+/CD44^+/CD62L^+$), effector memory $CD8^+$ T cells ($CD8^+$ TEM; $CD3^+/CD8^+/CD44^+/CD62L^-$) and effector memory $CD4^+$ T cells ($CD4^+$ TEM; $CD3^+/CD4^+/CD44^+/CD62L^-$). (B-E) After rechallenging the cured mice with MC38 tumor cells, the changes in the proportion of memory T cells in the mice's lymph nodes were systematically analyzed using flow cytometry. Results are shown as mean ± SEM. Statistical analyses performed by one-way ANOVA with Dunnett's multiple comparisons tests (ns, not significant; $^*p \leq 0.05$; $^{***}p \leq 0.001$). CR (Complete Response); LN (Lymph node).

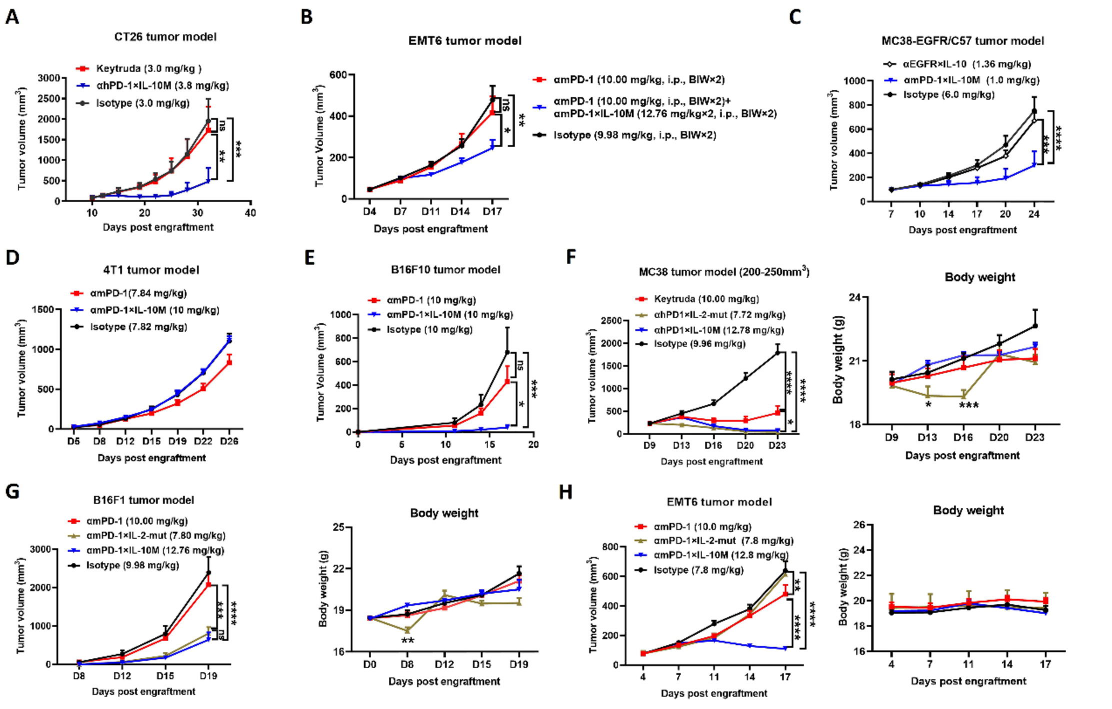

Figure S7. Anti-tumor efficacy of anti-PD-1×IL-10M in various tumor models. Related to Figure 6.

(A) Balb/c-hPD-1 mice were inoculated with 1×10^5 CT26 tumor cells. Tumor-bearing mice (n = 8/group) were intraperitoneally treated with indicated proteins on days 10, 13, 16 and 19. The tumor volume of mice was measured as indicated. (B) Balb/c mice were inoculated with 1×10^6 EMT6 tumor cells. Tumor-bearing mice (n = 8/group) were intraperitoneally treated with anti-mPD-1 proteins on days 4 and 7, anti-mPD-1×IL-10M proteins on days 11and 14. (C) C57BL/6 mice were inoculated with 1×10^6 MC38-EGFR tumor cells. Tumor-bearing mice (n = 8/group) were intraperitoneally treated with indicated proteins on days 7, 10, 14 and 17. (D) Balb/c mice were inoculated with 2×10^5 4T1 tumor cells. Tumor-bearing mice (n = 6/group) were intraperitoneally treated with indicated proteins on days 5, 8 and 12. (E) C57BL/6 mice were inoculated with 2×10^5 B16F10 tumor cells. Tumor-bearing mice (n = 7/group) were intraperitoneally treated with indicated proteins on days 0, 2 and 4. (F) C57BL/6-hPD1 mice were inoculated with 1×10^6 MC38 tumor cells. Tumor-bearing mice (n = 8/group) were intraperitoneally treated with indicated proteins on days 9, 13 and 16. The tumor volume and body weight of mice was measured as indicated. (G) C57BL/6 mice were inoculated with 2×10^5 B16F1 tumor cells. Tumor-bearing mice (n = 8/group) were intraperitoneally treated with indicated proteins on days 0, 2 and 4. The tumor volume and body weight of mice was measured as indicated. (H) Balb/c mice were inoculated with 1×10^6 EMT6 tumor cells. Tumor-bearing mice (n = 9/group) were intraperitoneally treated with indicated proteins on days 4, 7 and 11. The tumor volume and body weight of mice was measured as indicated. Results are shown as mean ± SEM. Statistical analyses performed by two-way ANOVA with Dunnett's multiple comparisons tests (ns, not significant, *p ≤ 0.05, **p ≤ 0.01, ***p ≤ 0.001, ****p ≤ 0.0001).
