# Supplementary material for: An innovative engineered IL-10 monomer strengthens T cell-mediated anti-tumor responses through anti-PD-1 cis-delivery
Source: Cell Rep Med. 2025 Dec 16;7(1):102515. doi: 10.1016/j.xcrm.2025.102515 (PMC12866158; doi:10.1016/j.xcrm.2025.102515)
Supplement: Document S2. Article plus supplemental information [file mmc2.pdf]

Cell Reports
Medicine

Article

An innovative engineered IL-10 monomer strengthens T cell-mediated anti-tumor responses through anti-PD-1 *cis*-delivery

Graphical abstract

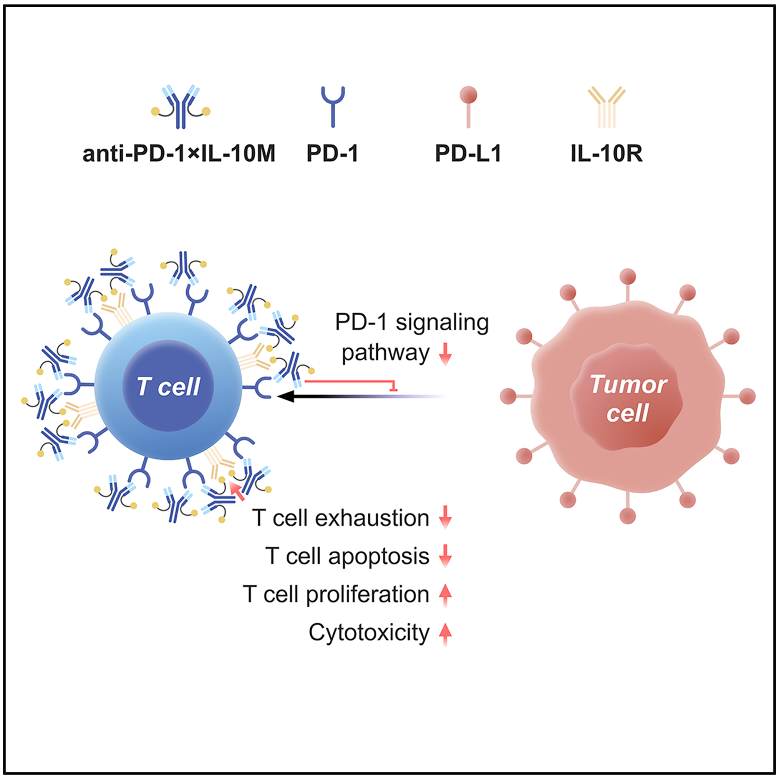

Authors

Ce Gu, Jian Guo, Chang Zhou, ..., Kongzhen Hu, Yongting Huo, Di Lu

Correspondence

ce.gu@fapon.com (C.G.),
cd.lu@fapon.com (D.L.)

In brief

The clinical application of IL-10 in cancer is constrained by severe hematological toxicity. Gu et al. design the fusion protein anti-PD-1×IL-10M, composed of anti-PD-1 and an attenuated IL-10 monomer (IL-10M), which exhibits favorable safety in cynomolgus monkeys and potent anti-tumor efficacy in various preclinical models.

Highlights

- Engineered IL-10M exhibits a significant reduction in activity and toxicity
- Anti-PD-1×IL-10M acquires enhanced bioactivity through anti-PD-1-mediated *cis*-binding
- Anti-PD-1×IL-10M reverses terminally exhausted $CD8^+$ T cells and shows potent antitumor efficacy
- Anti-PD-1×IL-10M has the potential to overcome the clinical safety limitations of IL-10

Gu et al., 2026, Cell Reports Medicine *7*, 102515
January 20, 2026 © 2025 Guangdong Fapon Biopharma Inc. Published by Elsevier Inc.
https://doi.org/10.1016/j.xcrm.2025.102515

CellPress

Cell Reports Medicine

CellPress
OPEN ACCESS

Article

An innovative engineered IL-10 monomer strengthens T cell-mediated anti-tumor responses through anti-PD-1 *cis*-delivery

Ce Gu,[1,5,*] Jian Guo,[1] Chang Zhou,[1] Peipei Hu,[1] Xiaodong Wu,[1] Jiaojiao Ding,[1] Xinxin Zhou,[1] Liao Zeng,[1] Wen Yu,[1] Yingye Ou,[1] Linhui Ye,[1] Mengying Liang,[1] Yue Huang,[1] Jiatian Li,[1] Zhe Zhang,[1] Wentao Deng,[1] Baiguang Ren,[1] Yingpei Zhang,[1] Li Wang,[1] Xuejiao Chen,[1] Yingxing Duan,[1] Zhe Han,[1] Yang Leng,[2] Hongxin Li,[3,4] Kongzhen Hu,[3,4] Yongting Huo,[1] and Di Lu[1,*]

[1]Guangdong Fapon Biopharma Inc, Dongguan, China
[2]Guangdong Jiyin Biotech Co. Ltd, Shenzhen, China
[3]Department of Nuclear Medicine, Nanfang Hospital, Southern Medical University, Guangzhou, China
[4]School of Pharmaceutical Sciences, Southern Medical University, Guangzhou, China
[5]Lead contact
*Correspondence: ce.gu@fapon.com (C.G.), cd.lu@fapon.com (D.L.)
https://doi.org/10.1016/j.xcrm.2025.102515

SUMMARY

Immune checkpoint blockade (ICB) therapy exerts anti-tumor efficacy mainly by activating intratumoral CD8$^+$ T cells but fails to re-activate terminally exhausted CD8$^+$ T cells. Interleukin-10 (IL-10) has been shown to directly expand and activate these cells and to exert a synergistic effect when combined with ICB. Nevertheless, the clinical application of IL-10 for cancer immunotherapy is restricted by severe hematological toxicity. Here, we design FP008 (anti-PD-1×IL-10M), a clinical-stage fusion protein composed of an anti-PD-1 antibody and an attenuated IL-10 monomer (IL-10M). Mechanistically, the activity and toxicity of IL-10M are significantly reduced, while its therapeutic benefits are enhanced through anti-PD-1-targeted enrichment and *cis*-activation. Anti-PD-1×IL-10M therapy displays robust anti-tumor activity in various mouse models, including those resistant to anti-PD-1 therapy, and exhibits promising safety in GLP toxicology studies in cynomolgus monkeys. Altogether, reinvigorating exhausted CD8$^+$ T cells in the tumor microenvironment through anti-PD-1×IL-10M represents a promising therapeutic strategy for overcoming anti-PD-1/L1-refractory solid tumors.

INTRODUCTION

ICB therapy, represented by anti-PD-1 antibodies, has brought breakthroughs to cancer treatment and achieved remarkable success in clinical practice.[1] However, most patients still face problems such as a low complete remission rate and susceptibility to drug resistance. Undoubtedly, these issues greatly limit the wide clinical application of ICB therapy.[2,3] To overcome resistance to PD-1 therapy, second-generation PD-1 therapeutics have emerged, including cytokine fusion proteins such as anti-PD-1×IL-2, anti-PD-1×IL-12, and anti-PD-1×IL-15, as well as bispecific antibodies like anti-PD-1/TIGIT and anti-PD-1/VEGF. Preclinical research and clinical trial data suggest that these second-generation anti-PD-1 molecules outperform traditional anti-PD-1 monoclonal antibodies in terms of tumor suppression.[4–7] They have great potential to replace anti-PD-1 and become the dominant clinical treatment option. The combination of anti-PD-1 and IL-2 is a popular area of clinical research.[8] For example, IBI363 by Innovent Biologics has achieved excellent efficacy in clinical practice. However, IL-2 can trigger activation-induced cell death (AICD) and further lead to T cell exhaustion.[9] Studies have shown that anti-PD-1 and IL-2 mainly act on PD-1$^+$ TCF-1$^+$ stem-like CD8$^+$ T cells but are ineffective against terminally exhausted CD8$^+$ T cells.[10] The clinical resistance to PD-1 therapy involves multiple complex aspects. Among them, the persistent presence of terminally exhausted T cells that are insensitive to PD-1 therapy is one of the key factors contributing to resistance.[11,12] Therefore, developing methods to reinvigorate exhausted CD8$^+$ T cells represents a crucial step toward achieving effective treatment, and conferring therapeutic benefits for PD-1/PD-L1-resistant patients.

Interleukin-10 (IL-10), a pleiotropic cytokine with potent anti-inflammatory properties, plays a pivotal role in immune regulation.[13] Natural IL-10 exists as a homodimer and specifically binds to IL-10Rα (also known as IL-10R1) to form an IL-10-IL-10Rα complex. Subsequently, this complex further binds to IL-10Rβ (IL-10R2) to form a hetero-hexamer that initiates downstream signaling.[14,15] Given that IL-10 plays a crucial regulatory role in restricting excessive inflammatory responses and preventing autoimmune diseases, it is often regarded as an immunosuppressive cytokine. However, IL-10 exerts a unique activating effect on CD8$^+$ T cells.[16] Mumm J et al. showed that PEGylated IL-10 (AM0010) can directly activate exhausted CD8$^+$ T cells by enhancing their proliferation and restoring their

© 2025 Guangdong Fapon Biopharma Inc. Published by Elsevier Inc.
This is an open access article under the CC BY license (http://creativecommons.org/licenses/by/4.0/).

function.[17] Guo Y et al. demonstrated that IL-10-Fc can directly enhance the expansion and effector functions of terminally exhausted $CD8^+$ T cells. Moreover, when combined with ICB immunotherapy, IL-10-Fc led to the complete eradication of established solid tumors in most experimental mouse models, resulting in durable cures.[18] Chang et al. reported that bacteria-induced IL-10 secretion by tumor-associated macrophages (TAMs) promotes intratumoral $CD8^+$ T cell function and proliferation, reactivates exhausted $CD8^+$ T cells, and strengthens anti-tumor immunity in multiple tumor models.[19] These studies have demonstrated the activation effect of IL-10 on exhausted $CD8^+$ T cells, providing additional insights and potential therapeutic strategies for tumor immunotherapy.

To date, AM0010 is the most extensively studied anti-tumor drug targeting IL-10.[20] It induced the production of high levels of Th1 and Th2 cytokines *in vivo*, thereby promoting the activation and expansion of tumor-specific $CD8^+$ T cells.[21] In a phase 1b clinical trial involving 111 patients, AM0010 demonstrated remarkable efficacy. When combined with PD-1 inhibitors, the objective response rate (ORR) was 41%, and the disease control rate (DCR) was 85% in patients with metastatic renal cell carcinoma (RCC). In patients with non-small-cell lung cancer (NSCLC), the ORR was 43% and the DCR was 82%. However, IL-10-related side effects generally limit the dosage of AM0010 to less than 20 μg/kg. Hematological adverse events such as severe anemia and thrombocytopenia occurred during treatment, which led many patients to further reduce the drug dosage or even discontinue treatment.[22,23] In phase 2 and 3 trials, the combination of AM0010 with PD-1 blockade or with chemotherapy did not significantly improve the ORR or progression-free survival (PFS).[24,25] The dose limitations and adverse reactions of AM0010 led to the failure of its subsequent clinical studies.

The severe hematological toxicity of IL-10 has become a pivotal bottleneck that restricts its extensive clinical application. In recent years, a series of innovative anti-tumor strategies based on IL-10 have emerged. Lemanbio has developed CAR-T cells that can secrete IL-10. The secreted IL-10 can effectively overcome and alleviate the problem of T cell exhaustion. It not only successfully induces complete tumor regression in multiple tumor models but also generates stem-like immune memory, thus achieving long-lasting immune protection.[26] SunHo Biopharma and Dingfu Biotarget fused cetuximab (anti-EGFR) with IL-10 to produce a bispecific fusion protein. Utilizing the specific targeting function of the antibody, IL-10 is delivered to tumors expressing EGFR, thereby reducing IL-10 systemic activity. Besides having an extended half-life without increasing toxicity, it has demonstrated superior anti-tumor activity compared to non-targeted IL-10 in mouse models.[27] The antibody-targeted specific delivery of IL-10 to the tumor microenvironment represents the current mainstream strategy for addressing the clinical toxicity issues of IL-10. However, the natural dimeric IL-10 in fusion proteins retains strong activity and still poses a potential risk of peripheral toxicity in clinical applications, especially at high doses. Therefore, how to further optimize the technical solution to reduce this potential risk has become the core issue that subsequent research urgently needs to overcome.

Josephson K et al. successfully constructed a stable IL-10 monomer (IL-10M) by inserting six amino acids (GGGSGG) between Asn116 and Lys117 of natural wild-type IL-10 (WT IL-10). The study revealed that the proliferative activity of IL-10M on B cells was significantly lower than that of WT IL-10.[28] Further research demonstrated that WT IL-10 forms a hetero-hexamer complex with IL-10Rα and IL-10Rβ receptors, while IL-10M forms a trimeric complex. This structural difference leads to a substantial weakening of the ability of the IL-10M to activate downstream signaling, and its functional activity is reduced.[29] Although the reduced functional activity of IL-10M may somewhat impede its efficacy, it shows great potential advantages in reducing hematological toxicity. Based on the above analysis, we believe that further structural design and optimization of IL-10M could effectively reduce the clinical toxicity of IL-10 while maintaining a certain level of IL-10 functional activity, thereby opening a new avenue for the development of anti-tumor drugs.

To this end, we have pioneered the development of a fusion protein, anti-PD-1×IL-10M, which is composed of anti-PD-1 fused with IL-10M. We engineered WT IL-10 into IL-10M, significantly reducing the functional activity of IL-10 and effectively weakening the phagocytic activity of macrophages toward red blood cells (RBCs). Meanwhile, anti-PD-1×IL-10M significantly enhances the binding and function of IL-10M to exhausted $CD8^+$ T cells in a *cis*-acting manner through the targeted enrichment property of the anti-PD-1 antibody moiety. Our research findings demonstrate that anti-PD-1×IL-10M can remarkably reverse the exhausted state of $CD8^+$ T cells. It not only significantly reduces the apoptosis of exhausted $CD8^+$ T cells and promotes cytokine secretion *in vitro* but also exhibits a potent anti-tumor effect in mice. Notably, in GLP toxicity studies in cynomolgus monkeys, anti-PD-1×IL-10M showed good tolerance and safety at doses as high as 10 mg/kg. Based on these characteristics, anti-PD-1×IL-10M shows considerable promise in delivering curative hope to patients with advanced solid tumors, especially those resistant to anti-PD-1 therapies.

RESULTS

WT IL-10 exerts anti-tumor efficacy *in vivo* and promotes erythrocyte phagocytosis *in vitro*

Blocking the PD-1/PD-L1 interaction in tumor immunotherapy can effectively relieve the inhibition of T cells, restoring their activity.[30] Simultaneously, IL-10 can promote the function of tumor-specific $CD8^+$ T cells.[16] Thus, we hypothesized a synergistic effect of PD-1/PD-L1 blockade and IL-10 in stimulating T cell responses. The results in CT26 tumor-bearing PD-1 humanized mice showed that WT IL-10-Fc and anti-hPD-1 alone had comparable anti-tumor effects, while the combination exhibited a more significant effect (Figure 1A), confirming WT IL-10's anti-tumor efficacy and synergy with anti-PD-1. However, AM0010 has exposed serious side-effect problems during clinical treatment, mainly manifested as severe anemia and thrombocytopenia.[24,25] The literature indicates that peripheral blood monocytes exhibit high-level expression of IL-10R, while other immune cells are relatively low.[31] To accurately identify the major cell subpopulations that may trigger these severe side effects, we evaluated the binding activity of WT IL-10-Fc to different human peripheral blood immune cells. Results showed that WT IL-10-Fc had the highest

CellPress
OPEN ACCESS

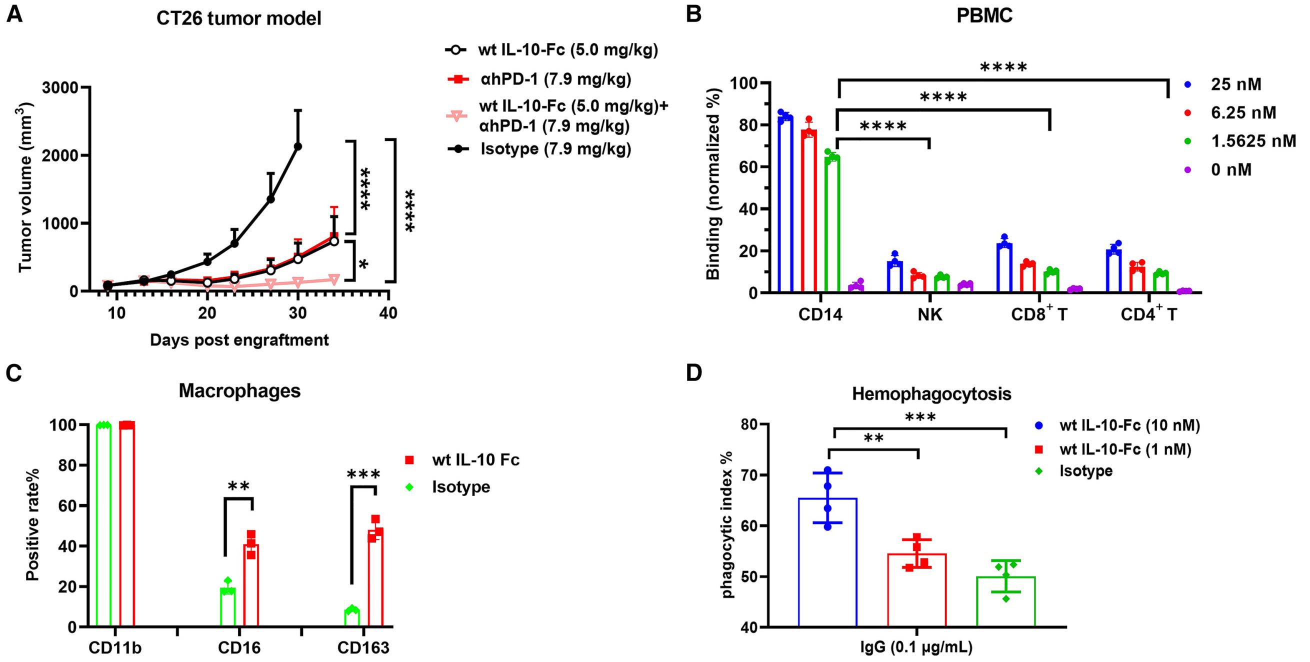

Figure 1. IL-10 exerts anti-tumor efficacy *in vivo* and promotes erythrocyte phagocytosis *in vitro*

(A) Balb/c-hPD-1 mice were inoculated with 1×10^5 CT26 tumor cells. Tumor-bearing mice ($n = 7$/group) were intraperitoneally treated with indicated proteins on days 9, 13, 16, and 20. The tumor volume of mice was measured as indicated. Statistical analyses were performed by two-way ANOVA with Dunnett's multiple comparisons tests (*$p \leq 0.05$; ****$p \leq 0.0001$).

(B) PBMCs were incubated with WT IL-10-Fc *in vitro*. Protein binding to $CD4^+$ T, $CD8^+$ T, NK, or CD14 cells was detected by flow cytometry (FCM). Statistical analyses were performed by one-way ANOVA with Dunnett's multiple comparisons tests (****$p \leq 0.0001$). Data are representative of three independent experiments performed on different donors.

(C) Monocytes were isolated from PBMCs and induced into macrophages using 50 ng/mL GM-CSF. The expression of CD11b, CD16, or CD163 was detected by FCM analysis. Statistical analyses were performed by unpaired Student's t tests. (**$p \leq 0.01$; ***$p \leq 0.001$). Data are representative of two independent experiments.

(D) RBCs were isolated from PBMCs and pre-incubated with IgG antibody for 30 min. The phagocytic activity of macrophages toward RBCs was detected by FCM analysis. Statistical analyses were performed by one-way ANOVA with Dunnett's multiple comparisons tests. (**$p \leq 0.01$; ***$p \leq 0.001$). Results are reported as the mean ± SEM. Data are representative of three independent experiments.

The hPD-1 knock-in humanized mouse is a model in which the mouse PD-1 gene is partially replaced with the human PD-1 gene using gene-editing technology, enabling the expression of human PD-1 protein.

binding level to $CD14^+$ monocytes (Figures 1B and S1A). This indicates that IL-10 preferentially binds to monocytes in the blood after administration, providing a key clue for exploring the root cause of IL-10-mediated severe side effects.

Research shows recombinant human IL-10 not only causes toxic side effects in cynomolgus monkeys but also induces significant enlargement of liver macrophages.[32] Based on this, we evaluated the effect of WT IL-10-Fc on the phagocytosis of RBCs by monocyte-derived macrophages. WT IL-10-Fc significantly upregulated macrophage surface phagocytic markers CD16 and CD163 (Figure 1C) and promoted the phagocytosis of IgG pre-treated RBCs in a concentration-dependent manner (Figure 1D). The flow cytometry plot of the macrophage-mediated RBC uptake process is shown in Figure S1B.

Collectively, these results indicate that anti-PD-1 antibody and WT IL-10 exhibit a synergistic effect in anti-tumor activity. However, WT IL-10 can significantly promote the phagocytosis of human RBCs by human macrophages, which may be a potential mechanism leading to the clinical hematological toxicity of IL-10. Evidently, modifying WT IL-10 to reduce its activity and hematological toxicity can significantly enhance its clinical potential.

Engineered IL-10M attenuates peripheral immune cell activation and toxicity

We inserted six amino acids (GGGSGG) between Asn116 and Lys117 of WT IL-10 and successfully engineered WT IL-10 into IL-10M (Figure 2A). As a typical immunosuppressive cytokine, IL-10 can effectively suppress the production of pro-inflammatory cytokines in peripheral blood mononuclear cells (PBMCs).[13] Therefore, the impact of IL-10M on human PBMC activity was investigated. Compared to WT IL-10-Fc, the inhibitory activity of IL-10M-Fc on the secretion of TNF-α by human PBMCs was significantly weaker (Figure 2B). Given that monocytes highly express IL-10R on their surface, the phosphorylation level of STAT3 in monocytes was detected. The results demonstrated that the phosphorylation level of STAT3 mediated by IL-10M-Fc was significantly lower (Figure 2C). Further, the effect of macrophages on the

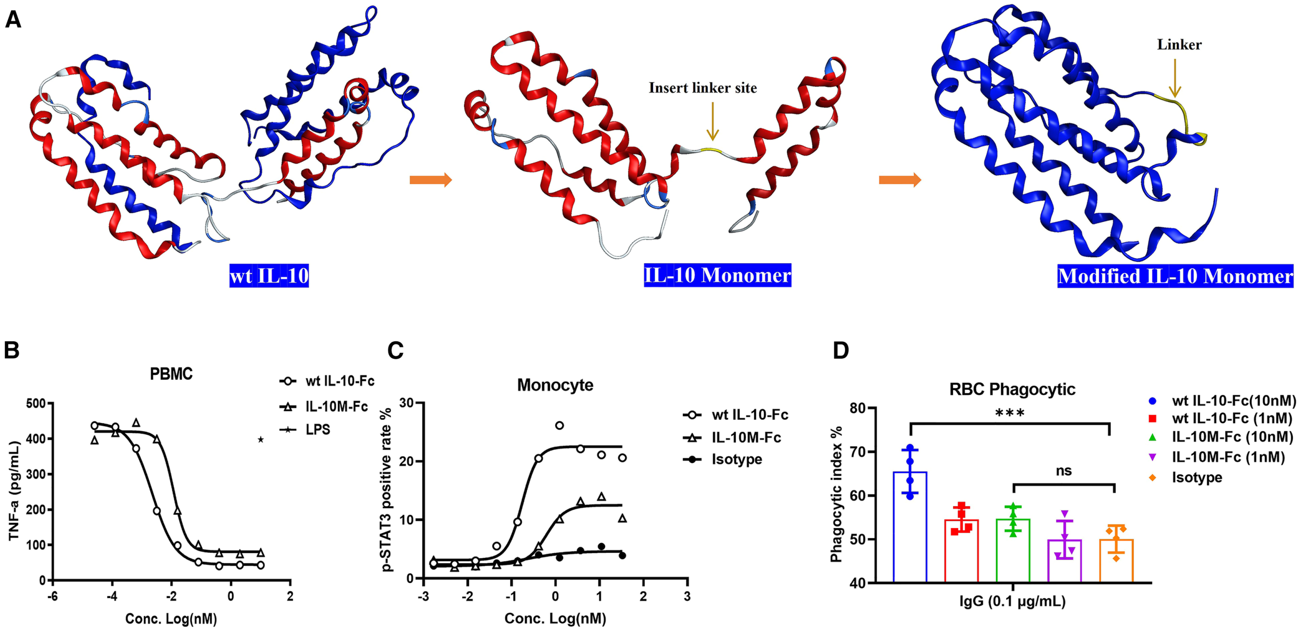

Figure 2. Engineered IL-10M attenuates peripheral immune cell activation and toxicity

(A) Schematic diagram of engineering WT IL-10 into IL-10M by inserting six amino acids (GGGSGG) between Asn116 and Lys117.

(B) PBMCs were incubated with a series of concentrations of WT IL-10-Fc or IL-10M-Fc for 6 h. Secretion of TNF-α in the supernatant was detected by ELISA. Data are representative of three independent experiments performed on different donors.

(C) Monocytes were isolated from PBMCs and incubated with indicated molecules for 0.5 h *in vitro*. The p-STAT3 level was detected by FCM analysis. Data are representative of two independent experiments performed on different donors.

(D) WT IL-10-Fc or IL-10M-Fc (1 nM and 10 nM) were incubated with monocyte-derived macrophages and pre-treated RBCs for 2 h *in vitro*. The phagocytic activity of macrophages toward RBCs was detected by FCM analysis. Statistical analyses were performed by one-way ANOVA with Dunnett's multiple comparisons tests (ns, not significant; $^{***}p \leq 0.001$). Results are reported as the mean ± SEM. Data are representative of three independent experiments.

phagocytic activity of RBCs was evaluated, and we found that IL-10M-Fc-mediated phagocytic activity was significantly weaker than that of WT IL-10-Fc at high concentration (Figure 2D).

In conclusion, the engineered IL-10M exhibits a remarkable reduction in activity. This provides a crucial theoretical basis for the potential of IL-10M to mitigate IL-10-related hematological toxicity in clinical applications.

Design and *in vitro* characterization of anti-PD-1×IL-10M

Previous studies have shown that PD-1 and IL-10R are highly expressed on tumor-infiltrating $CD8^+$ T cells.[6] We also observed IL-10R is highly expressed on $PD\text{-}1^+$ $TIM3^+$ exhausted $CD8^+$ T cells induced by anti-CD3/CD28 magnetic beads *in vitro* (Figure 3A). Based on the results that WT IL-10-Fc and anti-PD-1 exhibit a synergistic anti-tumor activity, we believe that anti-PD-1 antibody can serve as an ideal targeting molecule for the precise delivery of IL-10M to tumor-infiltrating $CD8^+$ T cells. Therefore, we designed an immune-cytokine fusion protein, anti-PD-1×IL-10M, which consists of anti-PD-1 antibody and IL-10M. IL-10M is linked to the C terminus of the anti-PD-1 antibody light chain via a (G4S)4G linker and the Fc region is a hinge-stabilized hIgG1 LALA mutant subtype (Figure 3B). This design was intended to enable the anti-PD-1 antibody to efficiently deliver IL-10M to intratumoral $PD\text{-}1^+$ $CD8^+$ T cells, rather than to peripheral NK or mononuclear cells, thereby effectively reducing peripheral immune cell-mediated toxicity and enhancing the functional activity of IL-10M to achieve better anti-tumor efficacy.

To evaluate the optimal conformation of the fusion protein, we also constructed an anti-PD-1×IL-10M (HC) fusion protein, in which IL-10M is linked to the C terminus of the anti-PD-1 antibody heavy chain (Figure S2A). *In vitro* reporter gene assay demonstrated that IL-10M linked to different parts of the antibody had similar activation activities on HEK-IL-10 reporter cells, which was significantly weaker than those of WT IL-10-Fc (Figure S2B). Meanwhile, anti-mPD-1×IL-10M (LC) showed significantly better anti-tumor efficacy in the CT26 tumor model (Figure S2C), suggesting that IL-10M may be more favorable for enhancing its functional activity via *cis*-acting mechanisms. Therefore, we ultimately selected the fusion protein with IL-10M linked to the light chain for further research. For the convenience of subsequent mechanism studies, the activities of anti-hPD-1×IL-10M and anti-mPD-1×IL-10M were evaluated *in vitro*. The results showed that the binding activity of both toward CHO-hIL-10R cells was comparable (Figure S2D), and the activity of blocking PD-L1 was also similar (Figure S2E), indicating that anti-mPD-1×IL-10M and anti-hPD-1×IL-10M possess comparable biological activities. Therefore, anti-mPD-1×IL-10M can serve as a surrogate for anti-hPD-1×IL-10M in the efficacy studies in WT mice.

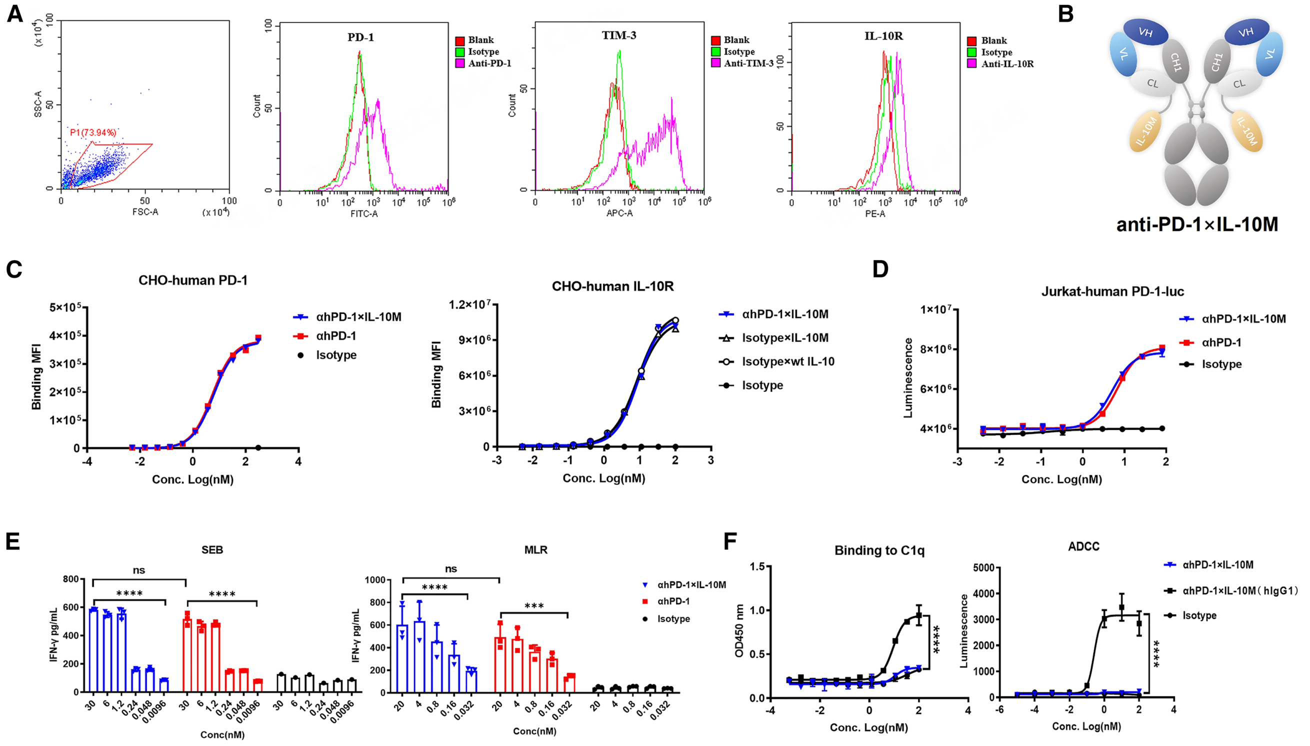

Figure 3. Design and *in vitro* characterization of anti-PD-1×IL-10M

(A) CD8+ T cells were isolated from PBMCs and induced by CD3/CD28 magnetic beads for 5 days *in vitro*, as the exhausted CD8+ T cells. The expression of PD-1, TIM-3, or IL-10R was detected by FCM analysis.

(B) Schematic diagram of anti-PD-1×IL-10M. IL-10M is linked to the C terminus of the anti-PD-1 antibody light chain via (G4S)4G.

(C) Binding of each indicated molecule to CHO-human PD-1 (left) or CHO-human IL-10R cells (right) was analyzed by FCM analysis. MFI means mean fluorescence intensity. Data are representative of three independent experiments.

(D) The bioactivity of the indicated proteins was detected in the reporter gene system.

(E) IFN-γ secretion in the supernatant was detected by ELISA in SEB (left) or MLR (right) experiments. Statistical analyses were performed by one-way ANOVA with Dunnett's multiple comparisons tests (ns, not significant; ***$p \leq 0.001$; ****$p \leq 0.0001$). Data are representative of two independent experiments performed on different donors.

(F) The Fc region is a hinge-stabilized hIgG1 mutant subtype. Binding of each indicated molecule to C1q was analyzed by ELISA (left), and ADCC activity was detected by reporter gene assay (right). Statistical analyses were performed by two-way ANOVA with Dunnett's multiple comparisons tests (****$p \leq 0.0001$). Results are reported as the mean ± SEM.

Then, the binding activity of both moieties of anti-hPD-1×IL-10M was evaluated. The results showed that the fusion protein had comparable binding activity to the self-developed parental humanized anti-hPD-1 and IL-10M control molecule, and the IL-10M had similar binding activity to WT IL-10 (Figure 3C). Subsequently, the functional activity of the anti-hPD-1 moiety in anti-hPD-1×IL-10M was evaluated. In the reporter gene system where CHO-OKT3-hPD-L1 and Jurkat-NFAT-luc-hPD-1 cells were co-incubated, anti-hPD-1×IL-10M induced comparable levels of reporter gene activity with the parental anti-hPD-1 antibody (Figure 3D). In addition, both the fusion protein and the parental anti-hPD-1 antibody showed a similar ability to promote IFN-γ secretion in SEB and MLR assays (Figure 3E). Therefore, anti-hPD-1×IL-10M retains functional activity comparable to that of the parental anti-hPD-1 antibody and can effectively exert anti-PD-1-mediated anti-tumor effects. Finally, Fc functional evaluation showed that compared with non-mutated hIgG1, the binding activity of anti-hPD-1×IL-10M to C1q protein was significantly reduced, and no luciferase signal was detected in the luciferase reporter gene assay (Figure 3F). These results indicate that anti-hPD-1×IL-10M does not mediate CDC or ADCC activity, thus effectively avoiding potential toxicity risks caused by the Fc region.

In summary, we have constructed an anti-PD-1×IL-10M fusion protein. It retains binding activity comparable to that of its parental molecules and preserves the functional activity of anti-PD-1. Moreover, the Fc region does not exhibit effector functions.

Anti-PD-1×IL-10M acquires enhanced bioactivity through anti-PD-1-mediated *cis*-binding

The proportion of PD-1+ and IL-10R+ CD8+ T cells within the tumor increased significantly as the tumor volume gradually increased in the MC38 tumor model. In particular, PD-1 and IL-10R were highly expressed on exhausted TOX+ CD8+ T cells (Figure S3A). Therefore, it is worth exploring whether anti-PD-1 antibody would help to enhance the binding activity and bioactivity of IL-10M on effector cells. We overexpressed

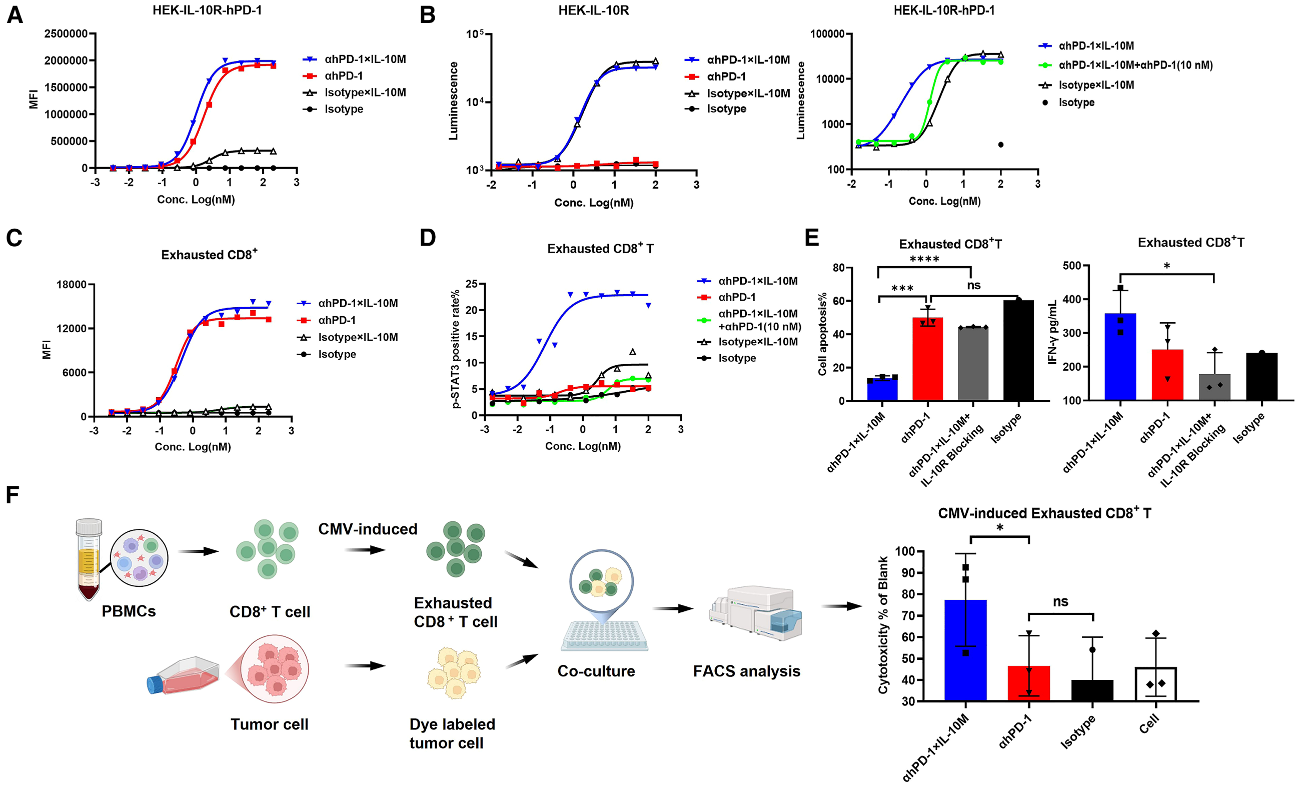

Figure 4. Anti-PD-1×IL-10M acquires enhanced bioactivity through anti-PD-1-mediated *cis*-binding

(A) Binding of each indicated molecule to HEK-IL-10R-hPD-1 reporter cells was detected by FCM analysis. MFI means mean fluorescence intensity. Data are representative of three independent experiments.

(B) The bioactivity of the indicated proteins was detected using HEK-IL-10R reporter cells (left) or HEK-IL-10R-hPD-1 reporter cells (right). For PD-1 blockade, 10 nM anti-PD-1 antibody was added. Data are representative of three independent experiments.

(C) Binding of each indicated molecule to exhausted CD8$^+$ T cells induced *in vitro*. Data are representative of three independent experiments performed on different donors.

(D) The bioactivity of the indicated proteins was detected using exhausted CD8$^+$ T cells; 10 nM anti-PD-1 antibody was added for PD-1 blockade. Data are representative of two independent experiments performed on different donors.

(E) Exhausted CD8$^+$ T cells were induced by CD3/CD28 magnetic beads for 5 days *in vitro*, then incubated with indicated proteins or an IL-10R inhibitor for 4 days. The apoptosis of exhausted CD8$^+$ T cells was detected by FCM analysis (left), and IFN-γ in the supernatant was detected by ELISA (right). Data are representative of two independent experiments performed on different donors.

(F) Schematic diagram of the killing of PANC.05.04 cells by CMV-induced exhausted CD8$^+$ T cells. CMV-induced exhausted CD8$^+$ T cells were co-cultured with PANC pre-loaded with pp65. The PANC.05.04 tumor cell killing efficiency was detected by FCM analysis. Data are representative of three independent experiments.

Results are reported as mean ± SEM. Statistical analyses were performed by paired Student's t tests. (ns, not significant; *$p \leq 0.05$, ***$p \leq 0.001$, ****$p \leq 0.0001$).

hPD-1 on HEK-IL-10R reporter gene cells to construct HEK-IL-10R-hPD-1 cells. Compared with the non-targeting control molecule Isotype×IL-10M, anti-hPD-1×IL-10M exhibited a significantly higher binding activity to HEK-IL-10R-hPD-1 cells, indicating that the anti-PD-1 moiety predominantly mediates binding to PD-1$^+$ effector cells (Figure 4A). In addition, anti-hPD-1×IL-10M and Isotype×IL-10M exhibited similar biological activities in HEK-IL-10R cells lacking PD-1 expression. However, the reporter gene activity of anti-hPD-1×IL-10M was significantly higher than that of Isotype×IL-10M in HEK-IL-10R-hPD-1 cells with overexpressed PD-1 (Figure 4B). Moreover, the reporter gene activity induced by anti-hPD-1×IL-10M was significantly reduced upon blockade with anti-hPD-1 antibody (Figure 4B). These results confirm that high levels of PD-1 expression on target cells can effectively enhance anti-hPD-1×IL-10M-mediated activation of the IL-10 signaling pathway.

Subsequently, the biological activity of anti-hPD-1×IL-10M was evaluated on in vitro-induced exhausted CD8$^+$ T cells, including its binding ability and induction of intracellular STAT3 phosphorylation. Binding assays revealed that the anti-PD-1 moiety of anti-hPD-1×IL-10M predominantly mediates binding to exhausted CD8$^+$ T cells (Figure 4C). Functional results showed that the phosphorylation level of STAT3 activated by anti-hPD-1×IL-10M was significantly higher than that of Isotype×IL-10M, and this effect was specifically blocked by anti-hPD-1 antibody (Figure 4D). This finding is highly consistent with the reporter gene assay results, collectively supporting the dual-action mechanism of anti-hPD-1×IL-10M. First, anti-hPD-1×IL-10M exhibits targeted enrichment, as the anti-PD-1 moiety specifically delivers IL-10M

to the surface of exhausted $CD8^+$ T cells via *cis*-binding to PD-1. Second, it enhances signaling, as high PD-1 expression on these cells significantly amplifies IL-10M-mediated downstream signaling. Together, these results demonstrate that anti-hPD-1×IL-10M preferentially activates $PD\text{-}1^+$ $CD8^+$ T cells through a *cis*-binding mechanism.

We further evaluated the functional activity of anti-hPD-1×IL-10M on exhausted $CD8^+$ T cells ($PD\text{-}1^+$ $TIM3^+$) induced by CD3/CD28 magnetic beads *in vitro*. Compared with anti-hPD-1 antibody, anti-hPD-1×IL-10M significantly reduced apoptosis and promoted IFN-γ secretion, and both of these effects can be significantly reversed by the IL-10R inhibitor (Figure 4E). In a cytotoxicity assay, CMV-induced exhausted $CD8^+$ T cells were co-cultured with PANC.05.04 cells; the expression of PD-L1 on PANC.05.04 cells is shown in Figure S3B. While anti-PD-1 showed limited killing activity, anti-hPD-1×IL-10M significantly enhanced tumor cell killing, and the addition of an IL-10R inhibitor significantly attenuated this killing-enhancing effect (Figures 4F and S3C). Collectively, these results indicate that anti-PD-1×IL-10M, via antibody-mediated *cis*-binding, forms a localized IL-10M-rich microenvironment on $PD\text{-}1^+$ $CD8^+$ T cells, thereby enhancing $CD8^+$ T cell function that depends on the IL-10-IL-10R signaling pathway. Notably, this effect is independent of PD-L1 expression, offering superior anti-tumor efficacy over anti-PD-1 antibodies and overcoming the limitations of conventional PD-1 blockade.

Anti-PD-1×IL-10M exerts anti-tumor efficacy dependent on intratumoral $CD8^+$ T cells

To assess whether anti-PD-1×IL-10M can accumulate in tumors *in vivo*, MC38 tumor-bearing mice were injected with zirconium-89 (^{89}Zr)-labeled anti-mPD-1×IL-10M or Isotype×IL-10M fusion proteins. PET imaging and tumor-to-muscle (T/M) uptake ratio showed significant tumor uptake of anti-mPD-1×IL-10M at 120 h, while Isotype×IL-10M showed minimal accumulation. The location of the tumor has been clearly marked with a red circle (Figure 5A). The biodistribution results further showed that the drug was mainly enriched in the liver, spleen, and tumor tissues (Figure 5B). *In vivo* evaluation of anti-tumor efficacy demonstrated that anti-mPD-1×IL-10M inhibited CT26 tumor growth in a dose-dependent manner (0.1–10 mg/kg; Figure S4A), outperforming anti-mPD-1 or IL-10-Fc alone (Figure 5C). Additionally, anti-mPD-1×IL-10M increased the proportion of $mCD45^+$ immune cells, especially $mCD3^+$ $CD8^+$ T cells, and decreased inhibitory $CD4^+$ $CD25^+$ $FOXP3^+$ Tregs (Figure 5D). In the MC38 model, anti-mPD-1×IL-10M inhibited tumor growth in a dose-dependent manner (0.1–1 mg/kg; Figure S4B) and demonstrated superior efficacy compared to anti-mPD-1 in large MC38 tumors (200–250 mm^3; Figure 5E). Flow cytometry revealed that anti-mPD-1×IL-10M significantly increased functional $IFN\text{-}\gamma^+$ $GZMB^+$ $CD8^+$ T cells within the tumor, whereas anti-mPD-1 did not (Figure 5F). Anti-mPD-1 promoted the differentiation of $CD8^+$ T cells into terminally exhausted $PD\text{-}1^{high}TOX^{high}$ cells, whereas anti-mPD-1×IL-10M prevented this differentiation. Furthermore, anti-mPD-1×IL-10M enhanced the ability of terminally exhausted $CD8^+$ T cells to secrete IFN-γ and GZMB (Figure 5F). These results suggest that anti-PD-1×IL-10M may be effective in treating tumors with limited or no response to anti-PD-1 by modulating the differentiation and function of terminally exhausted $CD8^+$ T cells.

We performed single-cell RNA sequencing of immune cells in MC38 tumors (Figure 5G). After treatment with anti-PD-1 antibody, the expression of functional molecules such as granzyme B, perforin, and IFN-γ, as well as activation markers, including CD27, IRF1, and PD-1, was markedly upregulated. Anti-PD-1 therapy relieves immune suppression and enhances cellular immune responses within the tumor microenvironment. However, the expression of exhaustion markers (Tox, Tim-3, CD39, and Lag-3) was also noticeably increased following anti-PD-1 treatment. Excessive activation of T cells might also trigger AICD, and indeed, apoptosis markers were also clearly upregulated after anti-PD-1 treatment. Anti-PD-1×IL-10M also promoted T cell activation in a manner similar to anti-PD-1. However, unlike anti-PD-1, anti-PD-1×IL-10M did not increase the expression of exhaustion or apoptosis markers, suggesting that the IL-10M moiety may help prevent exhaustion and promote T cell survival (Figure 5G). Based on marker expression, $CD8^+$ T cells were classified into seven functional subsets (Figure S4C). As shown in Figure 5H, the proportion of terminally exhausted T cells markedly increased following anti-PD-1 antibody treatment (50.6%) compared to the control group (23.2%). In contrast, anti-PD-1×IL-10M treatment reduced this proportion to 37.4%. Meanwhile, the proportion of effector T cells notably increased after anti-PD-1×IL-10M treatment (16.3%) compared to the PD-1 monotherapy group (4.7%), indicating that the IL-10M moiety may partially counteract PD-1-induced T cell exhaustion and increase the frequency of effector T cells. Pseudotime analysis further confirmed that anti-mPD-1 drove the transition from effector to terminally exhausted $CD8^+$ T cells, while anti-mPD-1×IL-10M counteracted this effect via IL-10M, limiting exhaustion (Figure S4D). These findings, consistent with TIL analysis, demonstrate anti-PD-1×IL-10M's unique mechanism in reversing terminal $CD8^+$ T cell exhaustion.

To clarify which immune cell types are mainly involved in the anti-tumor effect of anti-PD-1×IL-10M, we depleted NK cells, $CD4^+$ T cells, $CD8^+$ T cells, and macrophages using consumable antibodies during MC38 tumor treatment. Only $CD8^+$ T cell depletion abolished the therapeutic effect, while depletion of other cell types had minimal impact (Figures 5I and S4E), indicating that anti-PD-1×IL-10M primarily acts through $CD8^+$ T cells. In summary, these results demonstrate that the anti-PD-1×IL-10M fusion protein preferentially accumulates in tumor tissues via the anti-PD-1 antibody and exerts its effects primarily through $CD8^+$ T cells. It inhibits their differentiation into terminally exhausted cells while enhancing their effector function. As a result, anti-PD-1×IL-10M exhibits potent anti-tumor efficacy even in models that respond poorly to anti-PD-1 therapy.

Anti-PD-1×IL-10M exerts excellent anti-tumor efficacy in various tumor models and induces durable immune memory

We evaluated the anti-tumor efficacy of anti-PD-1×IL-10M in various tumor models. In the CT26 (Figure 6A) and MC38 (Figure 6B) models in hPD-1 knock-in mice, anti-hPD-1×IL-10M inhibited tumor growth in a dose-dependent manner

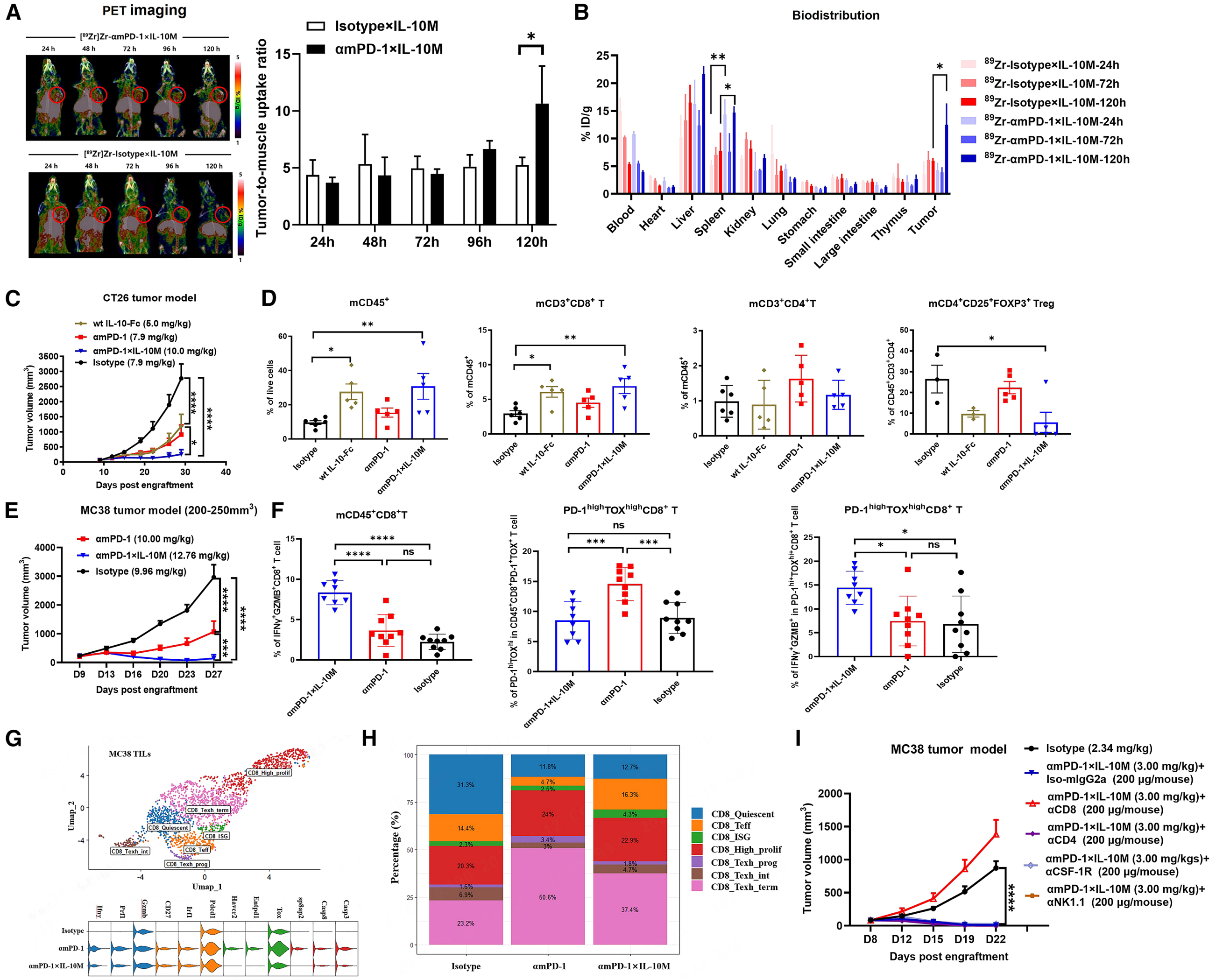

Figure 5. Anti-PD-1×IL-10M exerts anti-tumor efficacy dependent on intratumoral $CD8^+$ T cells

(A and B) Anti-mPD-1×IL-10M and Isotype×IL-10M fusion proteins were radiolabeled with zirconium-89 (^{89}Zr) and then intraperitoneally injected into MC38 tumor-bearing mice. PET imaging (left) and tumor-to-muscle uptake ratio (right) were performed at different time points to observe tumor uptake (A). Biodistribution of anti-mPD-1×IL-10M and Isotype×IL-10M in different tissues (B). Statistical analyses were performed by unpaired Student's *t* tests (**p* ≤ 0.05, ***p* ≤ 0.01).

(C) Balb/c mice were inoculated with 1 × 10^5 CT26 tumor cells. Tumor-bearing mice (*n* = 8/group) were intraperitoneally treated with indicated proteins on days 9, 12, and 15. The tumor volume of mice was measured as indicated. Statistical analyses were performed by two-way ANOVA with Dunnett's multiple comparisons test (**p* ≤ 0.05; *****p* ≤ 0.0001).

(D) The frequency of intratumoral $mCD45^+$, $mCD3^+$ $CD8^+$ T, $mCD3^+$ $CD4^+$ T, and $mCD4^+$ $mCD25^+$ $FOXP3^+$ Treg cells in the CT26 tumors after treatment, detected by FCM analysis. Statistical analyses were performed by one-way ANOVA with Dunnett's multiple comparisons test (**p* ≤ 0.05; ***p* ≤ 0.01).

(E) C57BL/6 mice were inoculated with 1 × 10^6 MC38 tumor cells. Tumor-bearing mice (*n* = 8/group) were intraperitoneally treated with indicated proteins on days 9, 13, and 16. The tumor volume of mice was measured as indicated. Statistical analyses were performed by two-way ANOVA with Dunnett's multiple comparisons test (****p* ≤ 0.001; *****p* ≤ 0.0001).

(F) The frequency of IFN-γ^+ $GZMB^+$ $CD8^+$ T cells, terminally exhausted PD-1^{high} TOX^{high} $CD8^+$ T, and IFN-γ^+ $GZMB^+$ terminally exhausted $CD8^+$ T cells in the MC38 tumors after anti-mPD-1×IL-10M and anti-mPD-1 antibody treatment, detected by FCM analysis. Statistical analyses were performed by one-way ANOVA with Dunnett's multiple comparisons test (ns, not significant; **p* ≤ 0.05; ****p* ≤ 0.001; *****p* ≤ 0.0001).

(G) C57BL/6 mice were inoculated with 5 × 10^5 MC38 tumor cells. UMAP showing the $CD8^+$ T cell clusters of $CD45^+$ cells treated with indicated proteins at equimolar concentrations.

(H) Percentage of $CD8^+$ T cell clusters treated with indicated proteins.

(I) C57BL/6 mice were inoculated with 3 × 10^5 MC38 tumor cells. Tumor-bearing mice (*n* = 6/group) were intraperitoneally treated with indicated proteins on days 8, 12, and 15. For cell depletion, mice were injected with 200 μg αCD4, αCD8, αNK, or αMo antibody 1 day before treatment and once every 3 days for three doses. The tumor growth curve of mice was monitored. Statistical analyses were performed by two-way ANOVA with Dunnett's multiple comparisons test (*****p* ≤ 0.0001).

Results are reported as mean ± SEM.

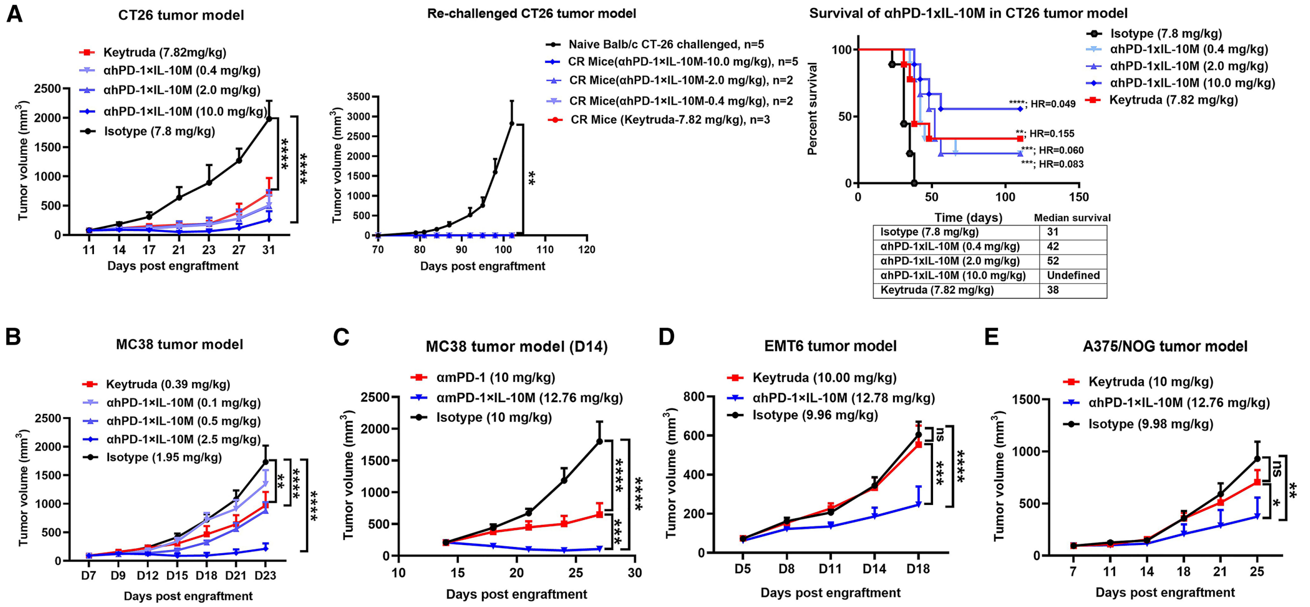

Figure 6. Anti-PD-1×IL-10M exerts excellent anti-tumor efficacy in various tumor models

(A) Balb/c-hPD-1 mice were inoculated with 1×10^5 CT26 tumor cells. Tumor-bearing mice ($n = 9$/group) were intraperitoneally treated with indicated proteins on days 11, 14, and 17; the tumor volume of mice was measured as indicated (left). Then, the mice with complete CT26 tumor regression were subcutaneously inoculated with 1×10^5 CT26 tumor cells. The tumor growth curve (middle) of the re-challenged mice and mice survival (right) were monitored over time. Naive Balb/c mice ($n = 5$/group) were challenged with 1×10^5 CT26 tumor cells as control.

(B) C57BL/6-hPD-1 mice were inoculated with 3×10^5 MC38 tumor cells. Tumor-bearing mice ($n = 9$/group) were intraperitoneally treated with indicated proteins on days 7, 10, and 13. The tumor volume of mice was measured as indicated.

(C) C57BL/6 mice were inoculated with 3×10^5 MC38 tumor cells. Tumor-bearing mice ($n = 9$/group) were intraperitoneally treated with indicated proteins on days 14, 18, and 21. The tumor volume of mice was measured as indicated.

(D) Balb/c-hPD-1 mice were inoculated with 1×10^6 EMT6 tumor cells. Tumor-bearing mice ($n = 8$/group) were intraperitoneally treated with indicated proteins on days 5, 8, and 11. The tumor volume of mice was measured as indicated.

(E) Humanized NOG mice were inoculated with 5×10^6 A375 tumor cells. Tumor-bearing mice ($n = 5$/group) were intraperitoneally treated with indicated proteins on days 7, 11, 14, 18, 21, and 25. The tumor volume of mice was measured as indicated.

Results are shown as mean ± SEM. Statistical analyses were performed by two-way ANOVA with Dunnett's multiple comparisons test (ns, not significant; $*p \leq 0.05$, $**p \leq 0.01$, $***p \leq 0.001$, $****p \leq 0.0001$).

(0.4–10.0 mg/kg for CT26; 0.1–2.5 mg/kg for MC38). After re-challenging the cured mice with CT26 tumor cells, all mice rejected the tumors and remained tumor free, indicating that anti-hPD-1×IL-10M can induce durable immune memory (Figure 6A). Furthermore, compared with anti-hPD-1×IL-10M, Keytruda showed limited or no efficacy at equivalent or higher doses (Figures 6A and S7A). To verify that anti-PD-1×IL-10M exerts anti-tumor effects by targeting exhausted $CD8^+$ T cells *in vivo*, we initiated drug administration 14 days after inoculating 3×10^5 MC38 tumor cells. At this time, the proportion of exhausted $CD8^+$ T cells was already relatively high in the TME. The results showed that anti-PD-1×IL-10M exhibited superior therapeutic efficacy compared with the anti-PD-1 antibody (Figure 6C). After rechallenging the cured mice with MC38 tumor cells, the changes in the proportion of memory T cells in the mice's spleens and lymph nodes were systematically analyzed using flow cytometry. Compared with the group of naive mice rechallenged with MC38 cells, both spleens and lymph nodes of mice had a significant increase in $CD8^+$ central memory T (TCM) cells ($CD8^+/CD44^+/CD62L^+$) frequency among total $CD8^+$ T cells in the treatment group, while there was no significant difference in $CD8^+$ effector memory T (TEM) cells ($CD8^+/CD44^+/CD62L^-$) (Figure S5). Meanwhile, both $CD4^+$ TEM and $CD4^+$ TCM were significantly increased in the treatment group (Figure S6). These results indicate that anti-hPD-1×IL-10M has the function of inducing durable immune memory. Furthermore, in the EMT6 model (Figure 6D) in hPD-1 knock-in mice and the PBMC-humanized NOG mouse model bearing A375 melanoma (Figure 6E), anti-hPD-1×IL-10M significantly suppressed tumor growth, whereas Keytruda showed no obvious effect. These findings demonstrate that IL-10M enhances the anti-tumor activity of anti-PD-1 when delivered as a T cell-targeted fusion protein.

The ability of anti-PD-1×IL-10M to overcome PD-1 resistance and exert anti-tumor efficacy was further verified in the EMT6 model. The results showed that anti-PD-1 antibody treatment alone remained ineffective throughout the entire experiment. In contrast, the group that received anti-PD-1 antibody twice first followed by the administration of anti-PD-1×IL-10M exhibited a significant tumor growth inhibitory effect (Figure S7B). This confirmed that anti-PD-1×IL-10M has the ability to overcome resistance to anti-PD-1 antibody. To clarify the advantages of the drug in this study over clinically investigational drugs, we compared the efficacy of anti-PD-1×IL-10M and anti-EGFR×IL-10 in mice bearing

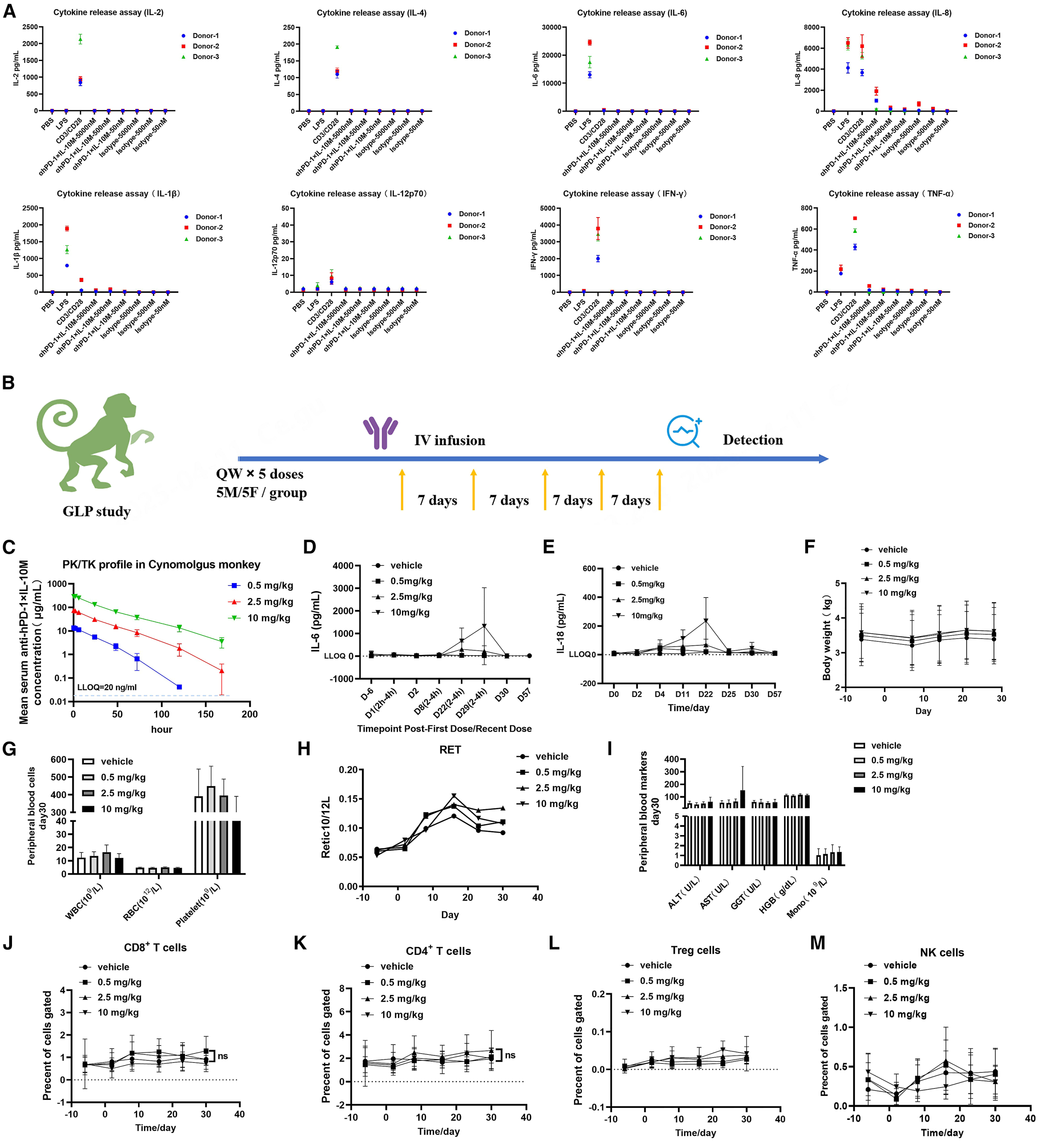

Figure 7. Anti-PD-1×IL-10M demonstrates favorable safety both in CRS study and GLP study

(A) PBMCs were isolated from three healthy human donors and incubated with negative control (PBS), positive control (LPS, anti-CD3/anti-CD28), or a series of concentrations of anti-PD-1×IL-10M and Isotype for 24 h *in vitro*. The secretion of IL-2, IL-4, IL-6, IL-8, IL-12p70, IFN-γ, TNF-α, and IL-1β in the supernatant was detected by LEGENDplex^{TM} kit (BioLegend).

(B) Schematic diagram of the 4-week repeated-dose toxicity study in cynomolgus monkeys. A total of 40 cynomolgus monkeys (n = 20/sex) were randomly assigned into four groups and received anti-PD-1×IL-10M once weekly at doses of 0 (vehicle), 0.5, 2.5, or 10 mg/kg by intravenous infusion for five doses.

(C) Anti-PD-1×IL-10M plasma concentration plotted up to 168 h after the first dose.

(D and E) Secretory levels of IL-6 and IL-18 cytokines (pg/mL) from animal plasma at the indicated study time points. Concentrations below the lower limit of quantification (LLOQ) are reported as 0 pg/mL.

(legend continued on next page)

MC38-EGFR tumors. The results showed that the anti-tumor efficacy of the former was significantly superior to that of the latter (Figure S7C). A possible explanation is that anti-EGFR×IL-10 is mainly enriched on the surface of tumor cells, and the IL-10 it carries activates $CD8^+$ T cells to exert anti-tumor effects but may also induce pro-tumor effects through macrophages and DCs, thereby affecting the overall therapeutic efficacy. However, anti-PD-1×IL-10M is mainly *cis*-enriched on the surface of tumor-infiltrating T cells and directly activates T cells to exert anti-tumor effects. In addition, we also evaluated the efficacy of anti-PD-1×IL-10M in low-T-cell-infiltration models. The fusion protein showed no obvious efficacy in the 4T1 model, while it could significantly inhibit tumor growth in the B16F10 model (Figures S7D and S7E). The above results indicate that anti-PD-1×IL-10M is not necessarily ineffective in cold tumors, and we are currently conducting in-depth mechanistic studies to uncover the molecular mechanisms.

Antibody-mediated targeting of cytokines to improve therapeutic efficacy and safety is a widely used strategy in drug development. The fusion protein IBI363, comprising an anti-PD-1 antibody and an IL-2 α-bias mutant, has shown encouraging progress in clinical trials. In this study, we generated two fusion proteins, anti-hPD-1×IL-2-mut (an IL-2 α-bias mutant from a patent) and anti-mPD-1×IL-2-mut, to evaluate the differences in efficacy and safety between anti-PD-1×IL-2-mut and anti-PD-1×IL-10M fusion proteins. In the MC38 model in hPD-1 knock-in mice and the B16F1 model, both fusion proteins showed superior anti-tumor activity compared to the parental antibody. However, anti-PD-1×IL-10M maintained stable body weight, whereas anti-PD-1×IL-2-mut treatment caused significant weight loss (Figures S7F and S7G). These findings suggest that the IL-10M fusion protein may have lower toxicity. In WT EMT6 models, anti-mPD-1 and anti-mPD-1×IL-2-mut had minimal effects, while anti-mPD-1×IL-10M significantly inhibited tumor growth without inducing weight loss (Figure S7H). In summary, anti-PD-1×IL-10M markedly enhanced anti-tumor efficacy across various tumor models compared to the limited or negligible effects of anti-PD-1. It also demonstrated comparable or superior efficacy to anti-PD-1×IL-2-mut, with the added advantage of potentially lower toxicity.

Anti-hPD-1×IL-10M demonstrates favorable safety both *in vitro* and *in vivo*

In the clinical application of immunomodulatory drugs, cytokine release syndrome (CRS) often leads to severe toxicity and poor prognosis. To evaluate the safety of anti-hPD-1×IL-10M, cytokine release was assessed in PBMCs from three healthy donors following treatment with this fusion protein. Anti-hPD-1×IL-10M only induced low IL-8 levels, while other cytokines (IL-2, IFN-γ, TNF-α, IL-4, IL-6, IL-12p70, and IL-1β) remained nearly undetectable (Figure 7A), suggesting a low risk of CRS-related toxicity. Based on the effects of anti-hPD-1×IL-10M observed in *in vitro* and *in vivo* experiments, we conducted a GLP toxicology study in cynomolgus monkeys. The monkeys received intravenous injections once weekly at 0.5, 2.5, and 10 mg/kg; a total of five doses were administered, followed by a 4-week recovery period (Figure 7B). Pharmacokinetic results showed that anti-hPD-1×IL-10M exhibited favorable linear pharmacokinetic characteristics at all the aforementioned three dose levels (Figure 7C). The results showed no significant increase in cytokine levels across any dose group; only the highest dose group (10 mg/kg) showed slight elevations in IL-6 and IL-18 levels, and these changes returned to normal after drug administration was completed (Figures 7D and 7E). Additionally, no dose-related changes in body weight were observed in either male or female animals (Figure 7F).

In terms of hematological parameters, anti-hPD-1×IL-10M did not alter the total number of peripheral blood white blood cells (WBCs) in cynomolgus monkeys nor did it cause a significant decrease in the counts of RBCs and platelets across any dose group (Figure 7G). Only the number of reticulocytes showed a mild increase with the elevation of dose (Figure 7H), suggesting no signs of severe anemia. Detection of liver function-related indicators revealed no abnormal changes in alanine aminotransferase (ALT), aspartate aminotransferase (AST), or γ-glutamyl transferase (GGT) in the peripheral blood of cynomolgus monkeys, indicating no signs of hepatotoxicity associated with this molecule (Figure 7I). Meanwhile, hemoglobin levels and monocyte counts also remained within the normal range (Figure 7I). Results of immune cell subset analysis showed that no dose-dependent changes in the proliferation frequency of $CD8^+$ T cells, $CD4^+$ T cells, or Treg cell subsets in the peripheral blood of cynomolgus monkeys were observed in any dose group. Only a slight decrease in the number of NK cells was seen in the highest dose group (10 mg/kg), and this change returned to normal after the end of drug administration (Figures 7J–7M).

In summary, the results of this study confirm the safety of *in vivo* administration of anti-hPD-1×IL-10M, with a maximum tolerated dose (MTD) of 10 mg/kg. Currently, we have initiated a clinical trial of anti-PD-1×IL-10M, and the first patient has been successfully enrolled. Meanwhile, the ongoing indication studies have covered a variety of malignant tumors, including renal cell carcinoma and non-small cell lung cancer.

DISCUSSION

IL-10 holds great potential in cancer immunotherapy by enhancing intratumoral exhausted $CD8^+$ T cell function. However, its short half-life and severe hematological toxicity limit its clinical use. To address these issues, we developed a bifunctional

(F) Male and female animal weights (kg) from day 6 to day 28 of anti-PD-1×IL-10M dosing in cynomolgus monkeys.
(G) Hematology analyses show the concentration of immune cells on day 30. Reported are WBCs, RBCs, and platelets.
(H) Changes in the number of reticulocytes (Retic) at the indicated study time points.
(I) Peripheral blood safety markers on day 30. Reported are alanine transaminase (ALT), aspartate aminotransferase (AST), gamma-glutamyl transferase (GGT), hemoglobin (HGB), and monocyte.
(J–M) Blood was collected at the indicated times to determine immune cell subset frequencies. Subsets reported are $CD8^+$ T (J), $CD4^+$ T (K), Treg (L), and NK cells (M). Statistical analyses were performed by one-way ANOVA with Dunnett's multiple comparisons test (ns, not significant).
Results are reported as mean ± SEM. LLOQ is indicated on several plots.

anti-PD-1×IL-10M fusion protein, which simultaneously blocks the PD-1/PD-L1 axis and activates the IL-10 pathway. The engineered IL-10M has reduced activity, but its function is enhanced by targeted enrichment and *cis*-activation with anti-PD-1, reducing side effects from systemic administration. *In vitro*, anti-PD-1×IL-10M reduces the apoptosis of exhausted CD8$^+$ T cells and enhances their cytotoxicity. TIL analysis and single-cell sequencing show that anti-PD-1×IL-10M prevents terminal exhaustion of CD8$^+$ T cells caused by anti-PD-1. *In vivo*, anti-PD-1×IL-10M demonstrates significant anti-tumor efficacy and good hematological safety in cynomolgus monkeys. Therefore, anti-PD-1×IL-10M may overcome the clinical ineffectiveness or resistance of anti-PD-1 therapy due to T cell exhaustion and has broad potential in tumor therapy.

Severe hematological toxicity in clinical trials led many patients to be unable to tolerate AM0010's side effects, requiring dose reduction or treatment discontinuation.[22–25] Previous toxicological studies of recombinant human IL-10 in cynomolgus monkeys, published by Pfizer, revealed potential risks.[32] Anemia symptoms were observed after administration, including a significant decrease in hemoglobin and platelets. Additionally, drug-related histopathological changes were noted, such as enlargement of liver macrophages and an increase in splenic plasma cells. The study suggested that anemia may be caused by extravascular hemolysis, where RBCs are recognized and phagocytosed by macrophages in the spleen and liver.[32] IL-10 has been shown to promote this process by enhancing the opsono-phagocytosis of IgG-coated RBCs by mononuclear macrophages. For example, IL-10 boosts phagocytosis of rituximab-opsonized B-CLL cells and enhances the phagocytosis of IgG-coated RBCs by monocyte-derived macrophages.[33–36] Based on these findings, we hypothesize that the increased splenic plasma cells in cynomolgus monkeys could be a result of IL-10 promoting B cell differentiation and IgG production. The enlargement of liver macrophages may be due to IL-10 promoting phagocytosis of IgG-coated RBCs. Together, these factors interact, ultimately causing severe anemia.

Given the severe hematological toxicity of WT IL-10 in clinical applications, the main strategy to address this is using antibodies to deliver IL-10 specifically to the tumor microenvironment. However, the IL-10 in this fusion exists in its dimeric form, which retains the activity to activate peripheral cells, posing a risk of peripheral toxicity. To overcome this, we engineered IL-10 into a monomeric form, IL-10M, and created a bifunctional immunecytokine, anti-PD-1×IL-10M. *In vitro* PBMC cytokine secretion and STAT3 phosphorylation assays confirmed the reduced activity of IL-10M. IL-10M showed weaker RBC phagocytic activity than WT IL-10, and anti-PD-1×IL-10M reduced hematological toxicity in GLP studies. This suggests that IL-10M may have lower hematological toxicity by reducing RBC phagocytosis by macrophages. Functionally, anti-PD-1×IL-10M is more effective in inhibiting exhausted CD8$^+$ T cell apoptosis and promoting target cell killing than anti-PD-1. In engineered cell lines and exhausted CD8$^+$ T cells, the anti-PD-1 moiety enhances IL-10M activation by promoting *cis*-binding, thus boosting its targeted activity. Single-cell sequencing shows that anti-mPD-1 drives effector CD8$^+$ T cells into terminal exhaustion, but anti-mPD-1×IL-10M effectively counters this, significantly reducing T cell exhaustion. In terms of anti-tumor efficacy, anti-PD-1×IL-10M shows superior efficacy compared to anti-PD-1 in tumor models and is at least as effective as anti-PD-1×IL-2-mut, with potentially lower toxicity. Safety evaluations, including *in vitro* cytokine release assays and *in vivo* hematological toxicity studies in cynomolgus monkeys, indicate that anti-PD-1×IL-10M is safe.

In conclusion, the anti-PD-1×IL-10M fusion protein offers significant innovation and potential in drug development. It addresses the hematological toxicity issue of IL-10-related antitumor drugs by using IL-10M, which has reduced activity. IL-10M is specifically enriched on exhausted PD-1$^+$ CD8$^+$ T cells in tumors via anti-PD-1 targeting, enhancing its activity while reducing non-specific binding during systemic administration. Anti-PD-1×IL-10M shows strong anti-tumor effects in various tumor models and provides long-term immune memory protection. GLP studies show that even at a high dose (10 mg/kg), anti-PD-1×IL-10M does not cause significant hematological toxicity in cynomolgus monkeys, indicating good safety and the potential to overcome IL-10's clinical safety limitations. It activates CD8$^+$ T cells, especially exhausted ones that are resistant to anti-PD-1, significantly reducing CD8$^+$ T cell exhaustion. This offers a promising treatment option for patients with drug resistance due to T cell exhaustion. Additionally, anti-PD-1×IL-10M, through anti-PD-1, activates CD8$^+$ T cells with high PD-1 expression. Even in patients with low PD-L1 expression, anti-PD-1×IL-10M can still activate T cells through IL-10M, avoiding the inefficacy of anti-PD-1. It holds promise for patients with drug resistance due to low or absent PD-L1 expression.

Limitations of the study

This study primarily utilized engineered human cell lines and primary human PBMCs to evaluate the *in vitro* activity of the candidate molecule. Future preclinical and clinical studies are required to further clarify the functional activity of anti-PD-1×IL-10M in human TILs. Additionally, given the proof-of-concept nature of this study, investigations into the *in vivo* mechanism of action underlying the candidate molecule's anti-tumor efficacy were all conducted using syngeneic WT murine tumor models. Due to interspecies differences, such models are unable to fully recapitulate the complex characteristics of human tumors. Meanwhile, this study did not verify the combined application strategy of anti-PD-1×IL-10M with T cell infiltration-promoting therapies; subsequent research can be carried out in more cold tumor models to further validate the efficacy and applicability of this combination strategy.

RESOURCE AVAILABILITY

Lead contact

Further information and requests for resources and reagents should be directed to and will be fulfilled by the lead contact, Ce Gu (ce.gu@fapon.com).

Materials availability

Fapon materials described in this manuscript may be made available to qualified, academic, noncommercial researchers through a materials transfer agreement (MTA). For questions about how Fapon shares materials, use the email address (cd.lu@fapon.com).

Data and code availability

- Single-cell RNA-seq data that support the findings of this study have been deposited into CNSA with accession number CNP0007809 and are publicly available as of the date of publication.

- This paper does not report the original code.
- Any additional information required to reanalyze the data reported in this paper is available from the lead contact upon request.

ACKNOWLEDGMENTS

We thank all lab members and the support from the Guangdong Jiyin Biotech Co. Ltd, Shenzhen, and Southern Medical University, Guangzhou. This work was supported by Guangdong Fapon Biopharma Inc, Dongguan.

AUTHOR CONTRIBUTIONS

C.G., J.G., C.Z., P.P.H., X.D.W., K.Z.H., and D.L. designed the studies. C.G., J.J.D., X.X.Z., L.Z., W.Y., Y.Y.O., L.H.Y., M.Y.L., Y.H., J.T.L., Z.Z., W.T.D., X.J.C., Y.X.D., Y.L., H.X.L., and Y.X.D. performed the experiments. C.G., J.G., C.Z., P.P.H., and Y.Y.O. analyzed the data. J.G. and Y.L. performed the bioinformatics analysis. D.L., X.D.W., B.G.R., Y.P.Z., L.W., Z.H., and Y.T.H. provided conceptual advice and guidance. C.G. drafted the manuscript with contributions from J.G., C.Z., P.P.H., Y.Y.O., and D.L.

DECLARATION OF INTERESTS

The main research content of this paper was completed by Guangdong Fapon Biopharma, Inc. D.L., Y.T.H., Y.B.Z., and J.Q.Y. are inventors on US patent US20250032583A1; "IL-10 Monomer Fusion Protein and Use Thereof." C.G., D.L., X.D.W., Y.Y.O., B.G.R., J.G., Z.H., J.J.D., W.T.D., and L.H.Y. are inventors on a pending CN patent application (WO2025077212A1; "Anti-PD1-IL10 Fusion Protein and Use Thereof").

STAR★METHODS

Detailed methods are provided in the online version of this paper and include the following:

- KEY RESOURCES TABLE
- EXPERIMENTAL MODEL AND STUDY PARTICIPANT DETAILS
  - Mice
  - Non-human primate
  - Cell lines
- METHOD DETAILS
  - Immune subset analysis by flow cytometry in mouse tumors
  - scRNA-seq analysis
  - Biodistribution analysis
  - HEK-IL-10R reporter assay
  - Measurement of p-STAT3 assay
  - Phagocytosis activity assay
  - TILs isolation and marker assay
  - Exhausted CD8^{+} T functional assay
  - *In vitro* CRS assay
  - Toxicity study in NHPs
- QUANTIFICATION AND STATISTICAL ANALYSIS

SUPPLEMENTAL INFORMATION

Supplemental information can be found online at https://doi.org/10.1016/j.xcrm.2025.102515.

Received: May 21, 2025
Revised: September 15, 2025
Accepted: November 17, 2025
Published: December 16, 2025

REFERENCES

1. Sharpe, A.H., and Pauken, K.E. (2018). The diverse functions of the PD1 inhibitory pathway. Nat. Rev. Immunol. *18*, 153–167. https://doi.org/10.1038/nri.2017.108.
2. Schoenfeld, A.J., and Hellmann, M.D. (2020). Acquired Resistance to Immune Checkpoint Inhibitors. Cancer Cell *37*, 443–455. https://doi.org/10.1016/j.ccell.2020.03.017.
3. Topalian, S.L., Hodi, F.S., Brahmer, J.R., Gettinger, S.N., Smith, D.C., McDermott, D.F., Powderly, J.D., Carvajal, R.D., Sosman, J.A., Atkins, M.B., et al. (2012). Safety, Activity, and Immune Correlates of Anti–PD-1 Antibody in Cancer. N. Engl. J. Med. *366*, 2443–2454. https://doi.org/10.1056/NEJMoa1200690.
4. Wu, J., Bloch, N., Chang, A.Y., Bhavsar, R., Wang, Q., Crawford, A., DiLillo, D.J., Vazzana, K., Mohrs, K., Dudgeon, D., et al. (2024). A PD-1-targeted, receptor-masked IL-2 immunocytokine that engages IL-2Rα strengthens T cell-mediated anti-tumor therapies. Cell Rep. Med. *5*, 101747. https://doi.org/10.1016/j.xcrm.2024.101747.
5. Shen, J., Zou, Z., Guo, J., Cai, Y., Xue, D., Liang, Y., Wang, W., Peng, H., and Fu, Y.-X. (2022). An engineered concealed IL-15-R elicits tumor-specific CD8+T cell responses through PD-1-cis delivery. J. Exp. Med. *219*, e20220745. https://doi.org/10.1084/jem.20220745.
6. Zou, Z., Shen, J., Xue, D., Li, H., Xu, L., Cao, W., Wang, W., Fu, Y.-X., and Peng, H. (2024). Anti-PD-1 cis-delivery of low-affinity IL-12 activates intratumoral CD8+T cells for systemic antitumor responses. Nat. Commun. *15*, 4701. https://doi.org/10.1038/s41467-024-49034-1.
7. Zhao, Y., Chen, G., Chen, J., Zhuang, L., Du, Y., Yu, Q., Zhuang, W., Zhao, Y., Zhou, M., Zhang, W., et al. (2023). AK112, a novel PD-1/VEGF bispecific antibody, in combination with chemotherapy in patients with advanced non-small cell lung cancer (NSCLC): an open-label, multicenter, phase II trial. eClinicalMedicine *62*, 102106. https://doi.org/10.1016/j.eclinm.2023.102106.
8. Hashimoto, M., Ramalingam, S.S., and Ahmed, R. (2024). Harnessing CD8 T cell responses using PD-1–IL-2 combination therapy. Trends Cancer *10*, 332–346. https://doi.org/10.1016/j.trecan.2023.11.008.
9. Marks-Konczalik, J., Dubois, S., Losi, J.M., Sabzevari, H., Yamada, N., Feigenbaum, L., Waldmann, T.A., and Tagaya, Y. (2000). IL-2-induced activation-induced cell death is inhibited in IL-15 transgenic mice. Proc. Natl. Acad. Sci. USA *97*, 11445–11450. https://doi.org/10.1073/pnas.200363097.
10. Hashimoto, M., Araki, K., Cardenas, M.A., Li, P., Jadhav, R.R., Kissick, H.T., Hudson, W.H., McGuire, D.J., Obeng, R.C., Wieland, A., et al. (2022). PD-1 combination therapy with IL-2 modifies CD8+ T cell exhaustion program. Nature *610*, 173–181. https://doi.org/10.1038/s41586-022-05257-0.
11. Miller, B.C., Sen, D.R., Al Abosy, R., Bi, K., Virkud, Y.V., LaFleur, M.W., Yates, K.B., Lako, A., Felt, K., Naik, G.S., et al. (2019). Subsets of exhausted CD8+ T cells differentially mediate tumor control and respond to checkpoint blockade. Nat. Immunol. *20*, 326–336. https://doi.org/10.1038/s41590-019-0312-6.
12. Ribas, A., and Wolchok, J.D. (2018). Cancer immunotherapy using checkpoint blockade. Science *359*, 1350–1355. https://doi.org/10.1126/science.aar4060.
13. Saraiva, M., and O'Garra, A. (2010). The regulation of IL-10 production by immune cells. Nat. Rev. Immunol. *10*, 170–181. https://doi.org/10.1038/nri2711.
14. Shouval, D.S., Ouahed, J., Biswas, A., Goettel, J.A., Horwitz, B.H., Klein, C., Muise, A.M., and Snapper, S.B. (2014). Interleukin 10 Receptor Signaling. Adv. Immunol. *14*, 177–210. https://doi.org/10.1016/B978-0-12-800267-4.00005-5.
15. Moore, K.W., De Waal Malefyt, R., Coffman, R.L., and O'Garra, A. (2001). Interleukin-10 and the Interleukin-10 Receptor. Annu. Rev. Immunol. *19*, 683–765. https://doi.org/10.1146/annurev.immunol.19.1.683.
16. Oft, M. (2019). Immune regulation and cytotoxic T cell activation of IL-10 agonists – Preclinical and clinical experience. Semin. Immunol. *44*, 101325. https://doi.org/10.1016/j.smim.2019.101325.
17. Mumm, J.B., Emmerich, J., Zhang, X., Chan, I., Wu, L., Mauze, S., Blaisdell, S., Basham, B., Dai, J., Grein, J., et al. (2011). IL-10 Elicits

IFNγ-Dependent Tumor Immune Surveillance. Cancer Cell *20*, 781–796. https://doi.org/10.1016/j.ccr.2011.11.003.

18. Guo, Y., Xie, Y.-Q., Gao, M., Zhao, Y., Franco, F., Wenes, M., Siddiqui, I., Bevilacqua, A., Wang, H., Yang, H., et al. (2021). Metabolic reprogramming of terminally exhausted CD8+ T cells by IL-10 enhances anti-tumor immunity. Nat. Immunol. *22*, 746–756. https://doi.org/10.1038/s41590-021-00940-2.
19. Chang, Z., Guo, X., Li, X., Wang, Y., Zang, Z., Pei, S., Lu, W., Li, Y., Huang, J.-D., Xiao, Y., and Liu, C. (2025). Bacterial immunotherapy leveraging IL-10R hysteresis for both phagocytosis evasion and tumor immunity revitalization. Cell *188*, 1842–1857.e20. https://doi.org/10.1016/j.cell.2025.02.002.
20. Salkeni, M.A., and Naing, A. (2023). Interleukin-10 in cancer immunotherapy: from bench to bedside. Trends Cancer *9*, 716–725. https://doi.org/10.1016/j.trecan.2023.05.003.
21. Naing, A., Infante, J.R., Papadopoulos, K.P., Chan, I.H., Shen, C., Ratti, N.P., Rojo, B., Autio, K.A., Wong, D.J., Patel, M.R., et al. (2018). PEGylated IL-10 (Pegilodecakin) Induces Systemic Immune Activation, CD8+ T Cell Invigoration and Polyclonal T Cell Expansion in Cancer Patients. Cancer Cell *34*, 775–791.e3. https://doi.org/10.1016/j.ccell.2018.10.007.
22. Naing, A., Papadopoulos, K.P., Autio, K.A., Ott, P.A., Patel, M.R., Wong, D.J., Falchook, G.S., Pant, S., Whiteside, M., Rasco, D.R., et al. (2016). Safety, Antitumor Activity, and Immune Activation of Pegylated Recombinant Human Interleukin-10 (AM0010) in Patients With Advanced Solid Tumors. J. Clin. Orthod. *34*, 3562–3569. https://doi.org/10.1200/JCO.2016.68.1106.
23. Naing, A., Wong, D.J., Infante, J.R., Korn, W.M., Aljumaily, R., Papadopoulos, K.P., Autio, K.A., Pant, S., Bauer, T.M., Drakaki, A., et al. (2019). Pegilodecakin combined with pembrolizumab or nivolumab for patients with advanced solid tumours (IVY): a multicentre, multicohort, open-label, phase 1b trial. Lancet Oncol. *20*, 1544–1555. https://doi.org/10.1016/S1470-2045(19)30514-5.
24. Spigel, D., Jotte, R., Nemunaitis, J., Shum, M., Schneider, J., Goldschmidt, J., Eisenstein, J., Berz, D., Seneviratne, L., Socoteanu, M., et al. (2021). Randomized Phase 2 Studies of Checkpoint Inhibitors Alone or in Combination With Pegilodecakin in Patients With Metastatic NSCLC (CYPRESS 1 and CYPRESS 2). J. Thorac. Oncol. *16*, 327–333. https://doi.org/10.1016/j.jtho.2020.10.001.
25. Hecht, J.R., Lonardi, S., Bendell, J., Sim, H.-W., Macarulla, T., Lopez, C.D., Van Cutsem, E., Muñoz Martin, A.J., Park, J.O., Greil, R., et al. (2021). Randomized Phase III Study of FOLFOX Alone or With Pegilodecakin as Second-Line Therapy in Patients With Metastatic Pancreatic Cancer That Progressed After Gemcitabine (SEQUOIA). J. Clin. Orthod. *39*, 1108–1118. https://doi.org/10.1200/JCO.20.02232.
26. Zhao, Y., Chen, J., Andreatta, M., Feng, B., Xie, Y.-Q., Wenes, M., Wang, Y., Gao, M., Hu, X., Romero, P., et al. (2024). IL-10-expressing CAR T cells resist dysfunction and mediate durable clearance of solid tumors and metastases. Nat. Biotechnol. *42*, 1693–1704. https://doi.org/10.1038/s41587-023-02060-8.
27. Qiao, J., Liu, Z., Dong, C., Luan, Y., Zhang, A., Moore, C., Fu, K., Peng, J., Wang, Y., Ren, Z., et al. (2019). Targeting Tumors with IL-10 Prevents Dendritic Cell-Mediated CD8+ T Cell Apoptosis. Cancer Cell *35*, 901–915.e4. https://doi.org/10.1016/j.ccell.2019.05.005.
28. Josephson, K., DiGiacomo, R., Indelicato, S.R., Iyo, A.H., Nagabhushan, T.L., Parker, M.H., and Walter, M.R. (2000). Design and Analysis of an Engineered Human Interleukin-10 Monomer. J. Biol. Chem. *275*, 13552–13557. https://doi.org/10.1074/jbc.275.18.13552.
29. Gorby, C., Sotolongo Bellón, J., Wilmes, S., Warda, W., Pohler, E., Fyfe, P.K., Cozzani, A., Ferrand, C., Walter, M.R., Mitra, S., et al. (2020). Engineered IL-10 variants elicit potent immunomodulatory effects at low ligand doses. Sci. Signal. *13*, eabc0653. https://doi.org/10.1126/scisignal.abc0653.
30. Liu, J., Chen, Z., Li, Y., Zhao, W., Wu, J., and Zhang, Z. (2021). PD-1/PD-L1 Checkpoint Inhibitors in Tumor Immunotherapy. Front. Pharmacol. *12*, 731798. https://doi.org/10.3389/fphar.2021.731798.
31. Saxton, R.A., Tsutsumi, N., Su, L.L., Abhiraman, G.C., Mohan, K., Henneberg, L.T., Aduri, N.G., Gati, C., and Garcia, K.C. (2021). Structure-based decoupling of the pro- and anti-inflammatory functions of interleukin-10. Science *371*, eabc8433. https://doi.org/10.1126/science.abc8433.
32. Rosenblum, I.Y., Johnson, R.C., and Schmahai, T.J. (2002). Preclinical Safety Evaluation of Recombinant Human Interleukin-10. Regul. Toxicol. Pharmacol. *35*, 56–71. https://doi.org/10.1006/rtph.2001.1504.
33. Michalak, S.S., Olewicz-Gawlik, A., Rupa-Matysek, J., Wolny-Rokicka, E., Nowakowska, E., and Gil, L. (2020). Autoimmune hemolytic anemia: current knowledge and perspectives. Immun. Ageing *17*, 38. https://doi.org/10.1186/s12979-020-00208-7.
34. Leidi, M., Gotti, E., Bologna, L., Miranda, E., Rimoldi, M., Sica, A., Roncalli, M., Palumbo, G.A., Introna, M., and Golay, J. (2009). M2 Macrophages Phagocytose Rituximab-Opsonized Leukemic Targets More Efficiently than M1 Cells In Vitro. J. Immunol. *182*, 4415–4422. https://doi.org/10.4049/jimmunol.0713732.
35. Capsoni, F., Minonzio, F., Ongari, A.M., Carbonelli, V., Galli, A., and Zanussi, C. (1995). IL-10 up-regulates human monocyte phagocytosis in the presence of IL-4 and IFN-γ. J. Leukoc. Biol. *58*, 351–358. https://doi.org/10.1002/jlb.58.3.351.
36. te Velde, A.A., de Waal Malefijt, R., Huijbens, R.J., de Vries, J.E., and Figdor, C.G. (1992). IL-10 stimulates monocyte Fc gamma R surface expression and cytotoxic activity. Distinct regulation of antibody-dependent cellular cytotoxicity by IFN-gamma, IL-4, and IL-10. J. Immunol. *149*, 4048–4052. https://doi.org/10.4049/jimmunol.149.12.4048.

STAR★METHODS

KEY RESOURCES TABLE

REAGENT or RESOURCE	SOURCE	IDENTIFIER
Antibodies		
Brilliant Violet 510TM anti-human CD8 Antibody (clone:SK1)	BioLegend	Cat#: 344732; RRID: AB_2564624
Brilliant Violet 421TM anti-human CD56 (NCAM) Antibody (clone: 5.1H11)	BioLegend	Cat#: 362552; RRID: AB_2566061
Brilliant Violet 421TM Rat IgG2a, κ Isotype Ctrl Antibody (clone: RTK2758)	BioLegend	Cat#: 400549; RRID: AB_2885080
Brilliant Violet 421TM anti-mouse CD279 (PD-1) Antibody (clone: 29F.1A12)	BioLegend	Cat#: 135221; RRID: AB_2562568
Brilliant Violet 510TM anti-mouse CD45 Antibody (clone: 30-F11)	BioLegend	Cat#: 103138; RRID: AB_2563061
Brilliant Violet 421TM anti-mouse CD3 Antibody (clone: 17A2)	BioLegend	Cat#: 100227; RRID: AB_10900227
Brilliant Violet 421TM anti-mouse CD62L Antibody (clone: MEL-14)	BioLegend	Cat#: 104435; RRID: AB_10900082
KIRAVIA Blue 520TM anti-mouse CD366 (Tim-3) Antibody (clone: RMT3-23)	BioLegend	Cat#: 119751; RRID: AB_3083269
KIRAVIA Blue 520TM Rat IgG2a, κ Isotype Ctrl Antibody (clone: RTK2758)	BioLegend	Cat#: 400575; RRID: AB_3097084
APC anti-human CD3 Antibody (clone: HIT3a)	BioLegend	Cat#: 300312; RRID: AB_314048
APC anti-mouse CD4 Antibody (clone: GK1.5)	BioLegend	Cat#: 100412; RRID: AB_312697
APC anti-human CD8 Antibody (clone: SK1)	BioLegend	Cat#: 344722; RRID: AB_2075388
APC anti-human CD11b antibody (clone: ICRF44)	BioLegend	Cat#: 301310; RRID: AB_314162
APC anti-human CD366 (Tim-3) Antibody (clone: A18087E)	BioLegend	Cat#: 364804; RRID: AB_2910409
APC Mouse IgG1, κ Isotype Ctrl Antibody (clone: MOPC-21)	BioLegend	Cat#: 400120; RRID: AB_2888687
PE anti-human CD210 (IL-10 R) Antibody (clone: 3F9)	BioLegend	Cat#: 308804; RRID: AB_314735
PE Rat IgG2a, κ Isotype Ctrl Antibody (clone: RTK2758)	BioLegend	Cat#: 400508; RRID: AB_326530
PE mouse IgG1, κ Isotype Ctrl Antibody (clone: MOPC-21)	BioLegend	Cat#: 400112; RRID: AB_2847829
PE anti-mouse IFN-γ Antibody (clone: XMG1.2)	BioLegend	Cat#: 505808; RRID: AB_315401
PE anti-mouse CD210 (IL-10 R) Antibody (clone:1B1.3a)	BioLegend	Cat#: 112705; RRID: AB_313518
PE anti-human CD16 Antibody (clone: 3G8)	BioLegend	Cat#: 302056; PRID: AB_2564139
PE anti-human CD163 Antibody (clone: GHI/61)	BioLegend	Cat#: 333606; PRID: AB_1134002
PE anti-mouse/human CD3 Antibody (clone: 17A2)	BioLegend	Cat#: 100206; PRID: AB_312663
PE anti-mouse/human CD44 Antibody (clone: IM7)	BioLegend	Cat#: 103007; PRID: AB_312958
FITC anti-mouse CD279 (PD-1) Antibody (clone: 29F.1A12)	BioLegend	Cat#: 135214; RRID: AB_10680238
FITC anti-human CD279 (PD-1) Antibody (clone: EH12.2H7)	BioLegend	Cat#: 329904; RRID: AB_940477
FITC Mouse IgG1, κ Isotype Ctrl Antibody (clone: MOPC-21)	BioLegend	Cat#: 400108; RRID: AB_326429
FITC anti-human CD14 Antibody (clone: HCD14)	BioLegend	Cat#: 325604; RRID: AB_830677
BD HorizonTM RB705 Rat Anti-Mouse CD8a (clone: 53–6.7)	BD pharmingen	Cat#: 570255
PerCP/Cyanine5.5 anti-human CD4 Antibody (clone: OKT4)	BioLegend	Cat#: 317428; RRID: AB_1186122

(Continued on next page)

Continued

REAGENT or RESOURCE	SOURCE	IDENTIFIER
PerCP/Cyanine5.5 anti-mouse CD8a Antibody (clone: 53–6.7)	BioLegend	Cat#: 100734; RRID: AB_2075238
Alexa Fluor® 700 anti-human/mouse Granzyme B Recombinant Antibody (clone: QA16A02)	BioLegend	Cat#: 372222; RRID: AB_2728388
Alexa Fluor® 488 anti-STAT3 Phospho (Tyr705) Antibody (clone: 13A3-1)	BioLegend	Cat#: 651006; RRID: AB_2572083
TruStain FcX™ PLUS (anti-mouse CD16/32) Antibody (clone: S17011E)	BioLegend	Cat#: 156603; RRID: AB_2783137
TOX Monoclonal Antibody (TXRX10), eFluor™ 660, eBioscience™ (clone: TXRX10)	Thermo Fisher	Cat#: 50-6502-82; RRID: AB_2574265
GoInVivo™ Purified anti-mouse CD4 Antibody (clone: GK1.5)	BioLegend	Cat#: 100461; RRID: AB_2616828
Human IgG antibody	Yeasen	Cat#: 36110ES60
Pacific Blue™ anti-human CD279 (PD-1) Antibody (clone: EH12.2H7)	BioLegend	Cat#: 329916; RRID: AB_2283437
Pacific Blue™ Mouse IgG1, κ Isotype Ctrl (clone: MOPC-21)	BioLegend	Cat#: 400131; RRID: AB_2923473
Purified anti-human CD210 (IL-10 R) Antibody	BioLegend	Cat#: 308802; RRID: AB_314734
**Chemicals, peptides, and recombinant proteins**		
wt IL-10-Fc	Fapon Biopharma	N/A
Anti-hPD-1	Fapon Biopharma	N/A
Anti-mPD-1	Fapon Biopharma	N/A
Keytruda	MSD	N/A
IL-10M-Fc	Fapon Biopharma	N/A
LPS	Sigma	Cat#: L6529
Anti-hPD-1×IL-10M	Fapon Biopharma	N/A
Anti-mPD-1×IL-10M (HC)	Fapon Biopharma	N/A
Anti-mPD-1×IL-10M (LC)	Fapon Biopharma	N/A
Isotype×IL-10M	Fapon Biopharma	N/A
Isotype×wt IL-10	Fapon Biopharma	N/A
Anti-hPD-1×IL-10M (hIgG1)	Fapon Biopharma	N/A
hPD-L1-mFc	Fapon Biopharma	N/A
mPD-L1-mFc	Fapon Biopharma	N/A
Anti-hPD-1×IL-2 mut	Fapon Biopharma	N/A
Anti-mPD-1×IL-2 mut	Fapon Biopharma	N/A
anti-NK1.1 antibody	Fapon Biopharma	N/A
anti-CD8 antibody (2.43)	Fapon Biopharma	N/A
anti-CSF-1R antibody	Fapon Biopharma	N/A
Cell Activation Cocktail (with Brefeldin A)	BioLegend	Cat#: 423304
Recombinant GM-CSF	T&L Biotechnology	Cat#: GMP-TL302-0100
Human CMV pp65 peptide	Sangon Biotech	Cat#: T510162-0001
Recombinant human IL-7	Sino Biological	Cat#: GMP-11821-HNAE
Recombinant mouse IL-2	T&L Biotechnology	Cat#: TLM820-0050
UltraComp eBeads™ Plus Compensation Beads	Invitrogen	Cat#: 01-3333-42
Native human C1q	abcam	Cat#: ab282858
Biotin Rabbit polyclonal to C1q	abcam	Cat#: ab48341
Propidium iodide Solution	BioLegend	Cat#: 421301
Annexin V Binding Buffer	BioLegend	Cat#: 422201
**Critical commercial assays**		
Zombie NIR™ Fixable Viability Kit	BioLegend	Cat#: 423105
Tumor Dissociation Kit, mouse	Miltenyi Biotec	Cat#: 130-096-730

(*Continued on next page*)

Continued		
REAGENT or RESOURCE	SOURCE	IDENTIFIER
FoxP3 fixation/permeabilization kit	Thermo Fisher	Cat#: 00-5521-00
Bright-Lumi™ Firefly Luciferase Assay Kit	Beyotime	Cat#: RG051M
BD Phosflow ™ Fix Buffer I	BD Biosciences	Cat#: 557870
Perm Buffer III	BD Biosciences	Cat#: 558050
MACS CD14 microbeads	Miltenyi Biotec	Cat#: 130-050-201
CellTrace™ Violet Cell Proliferation Kit	Thermo Fisher	Cat#: C34557
eBioscience™ Cell Proliferation Dye eFluor™ 670	Thermo Fisher	Cat#: 65-0840-90
ACK Lysing Buffer	Gibco	Cat#: A1049201
CD8 (TIL) MicroBeads, mouse	Miltenyi Biotec	Cat#: 130-116-478
CD8 MicroBeads, human	Miltenyi Biotec	Cat#: 130-045-201
Anti-human CD3/CD28 magnetic beads	T&L Biotechnology	Cat#: GMP-TL603-1000
Human IFN gamma Uncoated ELISA Kit	Invitrogen	Cat#: 88-7316-88
Legendplex™ Multiplex Assay Kit	BioLegend	N/A
TNF alpha Human Uncoated ELISA Kit	Invitrogen	Cat#: 88-7346-88
Deposited data		
Single-cell RNA-seq data	This paper	GEO: CNP0007809
Experimental models: Cell lines		
CT26	Shanghai Cell Bank	TCM37
MC38	PCRC	1101MOU-PUMC000523
B16F1	PCRC	1101MOU-PUMC000365
EMT6	ATCC	CRL-2755
A375	Shanghai Cell Bank	SCSP-533
B16F10	Shanghai Cell Bank	TCM36
4T1	Shanghai Cell Bank	TCM32
HEK-IL-10R	Genomeditech	GM-C07927
HEK-IL-10R-hPD-1	This paper	N/A
CHO-human PD-1	This paper	N/A
CHO-mouse PD-1	This paper	N/A
CHO-human IL-10R	This paper	N/A
CHO-OKT3-hPD-L1	This paper	N/A
Jurkat-human PD-1-luc	This paper	N/A
Experimental models: Organisms/strains		
BALB/c mice	Charles River	N/A
C57BL/6J mice	Charles River	N/A
BALB/c-hPD-1 mice	GemPharmatech	N/A
C57BL/6J-hPD-1mice	Biocytogen	N/A
NOG mice	Charles River	N/A
Software and algorithms		
CytoFLEX S	Beckman Coulter	https://www.beckmancoulter.cn/bls/BLS_cytoflexS/
Cell Ranger software (v.7.1.0)	10× Genomics Inc.	https://www.10xgenomics.com/
Seurat package (v.5.1.0)	N/A	https://satijalab.org/seurat/
SingleR (v.2.4.1)	N/A	https://github.com/SingleR-inc/SingleR
Doublet Finder package (v.2.0.3)	N/A	https://github.com/chris-mcginnis-ucsf/DoubletFinder
Monocle (v.2.30.1)	N/A	https://cole-trapnell-lab.github.io/monocle-release/
Inveon small-animal PET/CT scanner	N/A	https://www.siemens-healthineers.cn/molecular-imaging/pet-ct
xCELLigence RTCA DP	Agilent	https://www.agilent.com.cn/
GraphPad Prism v.8.3.0	GraphPad Software	https://www.graphpad.com/

EXPERIMENTAL MODEL AND STUDY PARTICIPANT DETAILS

Mice

Female BALB/c, C57BL/6J and NOG mice were purchased from Guangzhou Vital River Laboratory. BALB/c-hPD-1 and C57BL/6J-hPD-1 mice were purchased from GemPharmtech and Biocytogen Pharmaceuticals (Beijing) Co., Ltd. All animal experiments were conducted in strict compliance with the *Guide for the Care and Use of Laboratory Animals* from the National Institutes of Health. The protocol was approved by the Guangdong Fapon Pharmaceutical Experimental Animal Ethics Committee. All mice (6–7 weeks old) were maintained under specific pathogen-free conditions (40%–70% humidity and 20°C–26°C).

For tumor growth and treatment, unless otherwise indicated, different mouse strains were implanted with tumor cells resuspended in PBS by subcutaneous injection on the right flank. The following tumor cell numbers were used, MC38 (3×10^5), CT26 (1×10^5), B16F1 (2×10^5), EMT6 (1×10^6), MC38-EGFR (1×10^6), 4T1 (2×10^5), B16F10 (2×10^5) and A375 (5×10^6). When tumors were established, mice were randomized based on the tumor size and treated with indicated molecules and doses on the indicated days via intraperitoneal injection. For immune cell depletion studies, monoclonal antibodies (mAbs) used were as follows, mouse IgG2a isotype, anti-CD8, anti-CD4, anti-NK1.1, anti-CSF-1R. Depletion mAbs were administered by intraperitoneal injection 2 days before initiation of anti-PD-1×IL-10M dosing. Depletion mAbs were administered twice a week for a total of 4 doses. Anti-PD-1×IL-10M (3 mg/kg) was administered twice a week for a total of 3 doses.

Non-human primate

Cynomolgus monkeys (Macaca fascicularis) weighing 2.49 kg–5.35 kg (male) and 2.51 kg–3.6 kg (female) were used for PK and toxicity studies, housed in stainless steel cages in a controlled environment (40%–70% humidity and 18°C–26°C) on a 12 h light/dark cycle at various facilities where the in-life portion of the studies was conducted (JOINN Laboratories (Suzhou) Co., Ltd). The care or use of animals had been reviewed and approved by Institutional Animal Care and Use Committee (IACUC).

Cell lines

CT26, 4T1, B16F10 and A375 were purchased from Shanghai Cell Bank, MC38 and B16F1 were purchased from the Cell Resource Center, Peking Union Medical College (PCRC), EMT6 was purchased from ATCC. All cell lines were routinely tested for mycoplasma contamination. The MC38-EGFR cell line was constructed in-house. B16F10, MC38 and MC38-EGFR cells were cultured in DMEM containing 10% inactivated fetal bovine serum (FBS), 1% penicillin-streptomycin, 1% sodium pyruvate and 1% glutamine. B16F1 cells were cultured in DMEM containing 10% inactivated FBS, 1% penicillin-streptomycin. CT26 and 4T1 cells were cultured in RPMI 1640 containing 10% inactivated FBS, 1% penicillin-streptomycin. EMT6 cells were cultured in Waymouth's MB 752/2 Medium with 15% heat-inactivated FBS and 2mM L-glutamine. A375 cells were cultured in DMEM containing 10% inactivated FBS, 1% penicillin-streptomycin.

PK136 cells were purchased from National Collection of Authenticated Cell Cultures, and cultured in DMEM containing 10% FBS, 1% penicillin-streptomycin. anti-NK1.1 antibody (PK136) was purified from the supernatant of PK136 cells. anti-CD4 antibody (GK1.5) was purchased from Biolegend. anti-CD8 antibody (2.43) and anti-CSF-1R antibody were produced in-house according to patents. All fusion proteins were produced in-house.

METHOD DETAILS

Immune subset analysis by flow cytometry in mouse tumors

BALB/c mice were subcutaneously inoculated with CT26 cells. Mice were randomized into groups (n = 8 per group) and dosed every 3 days when tumors reached an average volume of 200–250 mm^3, tumors were digested and fixed broken, cells were first stained with live/dead staining with Zombie NIR Fixable Viability kit (Biolegend, 423105) and Fc receptors were blocked with Fc block TruStain FcX PLUS (anti-mouse CD16/32) antibody (Biolegend, 156604). Cell surface proteins were stained with indicated antibodies (anti-mouse CD45, CD3, Treg, and CD8 from Biolegend).

C57BL/6J mice were subcutaneously inoculated with MC38 cells. Mice were randomized into groups (n = 9 per group) and dosed every 3 days when tumors reached an average volume of 450–500 mm^3. After two doses, Tumors were digested into a single-cell suspension using Tumor Dissociation Kit (Miltenyi, 130-096-730) and passed through a 70-mum filter. For intracellular cytokine detection, the suspension was incubated with cell activation cocktail containing brefeldin A (Biolegend, 423304) at 37°C for 4 h according to the manufacturer's protocol. Cells were first stained with live/dead staining with Zombie NIR Fixable Viability kit (Biolegend, 423105) and Fc receptors were blocked with Fc block TruStain FcX PLUS (anti-mouse CD16/32) antibody (Biolegend, 156604). Cell surface proteins were stained with indicated antibodies. Then, after fixation and lysis using FoxP3 fixation/permeabilization kit (Thermo Fisher Scientific, 00-5521-00), intracellular proteins were stained. Flow cytometry was performed using a CytoFLEX S (Beckman Coulter), and data were analyzed with CytExpert software. The following fluorochrome-conjugated antibodies were employed for surface and intracellular immunostaining. CD45-BV510 (Biolegend, 103138), CD8-RB705 (BD pharmingen, 570255), PD-1-FITC (Biolegend, 135214), TOX-eF660 (Thermo Fisher Scientific, 50-6502-82), TCF-1-BV421 (BD pharmingen, 566692), IFNγ-PE (Biolegend, 505808) and Granzyme B-AF700 (Biolegend, 372222).

CellPress
OPEN ACCESS

scRNA-seq analysis

MC38 cells (5×10^5 per mouse) were implanted in C57BL/6 mice. When the average tumor volume reached about 270 mm^3, the mice were randomly assigned and intraperitoneally injected with anti-mPD-1×IL-10M, anti-mPD-1, and Isotype at equimolar concentrations, with corresponding doses of 12.8 mg/kg, 10 mg/kg, and 10 mg/kg, respectively, twice a week. One day after the third dose, tumors were harvested and digested. Subsequntly, live $CD45^+$ cells were isolated by flow cytometry and sent to Azenta Life Sciences for scRNA-seq library construction and sequencing using the 10× Genomics platform.

scRNA-seq data were processed using Cell Ranger software (v.7.1.0). Downstream analyses were performed in R using the Seurat package (v.5.1.0). Cells in which fewer than 300 genes were detected and in which mitochondrially encoded transcripts constituted greater than 25% of the total library were excluded from downstream analysis. Genes detected in fewer than 5 cells across the dataset were also excluded. To integrate single cells from different individuals in a shared low-dimensional space, we utilized Harmony (v.1.2.3) integrated analysis perform batch effect correction and normalization.

We applied SingleR (v.2.4.1) for cell type annotation and identified T cells, then isolated $CD3^+$ $CD8^+$ $CD4^-$ T cells ($CD8^+$ T cells) for further analysis. A standardized analysis was performed on the $CD8^+$ T cell data including data preprocessing, feature selection, normalization, cell cycle scoring, PCA, UMAP dimensionality reduction, neighbor finding, and clustering analysis by Seurat. The number of principal components was set to 50, the number of features was set to 4000, the "PCA" dimensionality reduction method was used. The Doublet Finder package (v.2.0.3) was used for doublet detection. The clusters of $CD8^+$ T cells were annotated by known markers as shown in (Figure S4C). The clusters "CD8_Quiescent," "CD8_Teff," "CD8_High_prolif," and "CD8_Texh_term" were further selected for pseudo time trajectory analysis using Monocle (v.2.30.1). The results were visualized using Ggplot2 R package (v.3.5.1).

Biodistribution analysis

The bifunctional chelating agent *p*-isothiocyanatobenzyl-desferrioxamine (DFO-Bz-NCS) was conjugated with lysine residues of anti-mPD-1×IL-10M and Isotype×IL-10M to produce DFO-*anti*-mPD-1×IL-10M and Isotype×IL-10M following the literature method with minor modifications[x]. The radiolabeled conjugates were purified by PD-10 column using 0.9% NaCl. The molar activity and purity of the radiolabeled antibodies were determined by radio-HPLC using a MAbPac RP column (3.0 × 100 mm 4μm).

Mice bearing MC38 xenografts were intravenously injected with 2–2.8 MBq (0.4 nmol) of [^{89}Zr] Zr-DFO- anti-mPD-1×IL-10M ($n = 3$) or [^{89}Zr] Zr-DFO-Isotype×IL-10M ($n = 3$). They underwent scans with acquisition times ranging from 30 min at 24-, 48-, 72-, and 96- or 120-h post-injection. PET studies were conducted using Inveon small-animal PET/CT scanner (Siemens, German). For biodistribution studies, mice received intravenous injections of 2.6–3.0 MBq (0.4 nmol) of [^{89}Zr] Zr-DFO-*anti*-mPD-1×IL-10M or [^{89}Zr] Zr-DFO-Isotype×IL-10M. Mice (n = 4/timepoint) were euthanized by cervical dislocation at 24, 72 and 120 h. Organs (blood, heart, lungs, liver, spleen, stomach, small intestines, large intestines, kidneys, tumor and thymus) were collected in pre-weighed tubes. The radioactivity of probe distribution was assessed using a γ-counter, and presented as the percentage of injected dose per gram of tissue (%ID/g) for all organs.

HEK-IL-10R reporter assay

HEK-IL-10R cell line was transduced with lentivirus encoding the human PD-1 gene, and the HEK-IL-10R-hPD-1 cell line was obtained. HEK-IL-10R cell line and HEK-IL-10R-hPD-1 cell line were used to detect the activity of IL-10 fusion protein *in vitro*. 2.5×10^4 cells were incubated with different concentrations of proteins for 16 h in 5% CO_2 at 37°C, then 100 μL Bright-Lumi (Beyotime, RG051M) was added and incubated for 5–10 min. The reporter gene activity was assessed by detecting luminescent signals on Ensight microplate reader. For PD-1 blockade, 10 nM anti-hPD-1 was added into the incubation system of HEK-IL-10R-hPD-1 cells and anti-hPD-1×IL-10M fusion proteins.

Measurement of p-STAT3 assay

In vitro induced exhausted $CD8^+$ T cells or monocytes isolated from PBMCs were incubated with gradient dilutions of different antibodies at 37°C for 30 min. Then add pre-heated BD Phosflow Fix Buffer I (BD Biosciences, 557870), fixed at 37°C for 15 min. After washing, pre-cooled Perm Buffer III (BD Biosciences, 558050) was added for permeabilized at 4°C for 30 min. Following another wash, cells were stained with Alexa Fluor 488 anti-STAT3 Phospho (Tyr705) Antibody (Biolegend, 651006) and analyzed by Flow Cytometry.

Phagocytosis activity assay

Monocytes were isolated from PBMCs using MACS CD14 microbeads (Miltenyi Biotec, 130-050-201). 2.5×10^5 cells/mL monocytes were added with 50 ng/mL GM-CSF (Beijing T&L Biotechnology Co., Ltd, GMP-TL302-0100) for 3 days in 5% CO_2 at 37°C, and then supplement 50 ng/mL GM-CSF for another 3 days to obtain macrophages. After washing, all cells were gently scraped off with a cell scraper and stained with the CellTrace Violet Cell Proliferation Kit (Thermo Fisher, C34557). Cells were seeded into 96-well plates and incubated with gradient dilutions of different antibodies for 24 h in 5% CO_2 at 37°C. Subsequently, relevant surface markers were analyzed by Flow Cytometry.

Allogeneic RBCs and plasma were obtained by density gradient centrifugation. The RBCs were stained with eBioscience Cell Proliferation Dye eFluor 670 (Thermo Fisher, 65-0840-90) and pre-incubated with IgG antibody (Yeasen, 36110ES60) diluted in plasma

for 30 min in 5% CO_2 at 37°C. Subsequently, co-incubated with the macrophages for 6 h in 5% CO_2 at 37°C, and analyzed by Flow Cytometry.

TILs isolation and marker assay

Freshly isolated tumor tissues were cut into small pieces and resuspended in digestion buffer. The tumors were digested at 37°C for 40 min, and then passed through a 70-μm cell strainer. The cells were washed with complete DMEM medium and RBCs were removed using ACK Lysing Buffer (Gibco, A1049201) to obtain a single-cell suspension. For marker detection, single-cell suspension was incubated with TruStain FcX PLUS (anti-mouse CD16/32) Antibody (Biolegend, 156603) at 4°C in the dark for 15 min. Then, live-dead cell staining and surface marker staining were carried out according to the staining protocol. To detect intracellular TOX, cells were fixed, permeabilized, and stained with anti-mouse TOX antibody. Data were analyzed by Flow Cytometry.

Exhausted $CD8^+$ T functional assay

$CD8^+$ T cells were isolated from PBMCs using CD8 MicroBeads, human (Miltenyi Biotec, 130-045-201) according to the manufacturer's protocol. Anti-human CD3/CD28 magnetic beads (Beijing T&L Biotechnology Co., Ltd, GMP-TL603-1000) were added in a 1:1 ratio of 1×10^6 cells/mL $CD8^+$ T cells, and cultured in 5% CO_2 at 37°C for 5 days. The magnetic beads were removed, and the relevant surface marker and binding activity was detected. The stimulating activated $CD8^+$ T as exhausted $CD8^+$ T cells. Meanwhile, apoptosis and cytokine detection were further performed. The specific operations were as follows: Different antibody gradient dilutions were added to a 96-well plate pre-coated with PD-1 protein and incubated at 37°C for 60 min 1×10^6 cells/mL exhausted $CD8^+$ T cells mixed with CD3&CD28 magnetic beads at a ratio of 1:1 and cultured for another 4 days. The secretion level of human IFN-γ in the supernatant was detected according to the manufacturer's protocol (Invitrogen, 88-7316-88). The cells were double-stained with APC annexin-V and PI, and analyzed by Flow Cytometry.

2×10^6 cells/mL PBMCs were cultured in RPMI-1640 medium containing 1 μg/mL Human CMV pp65 peptide (Sangon Biotech, T510162-0001), 2 ng/mL hIL-2, and 10 ng/mL hIL-7 (Sino Biological, GMP-11821-HNAE) for 2 days in 5% CO_2 at 37°C. Subsequently, 2 ng/mL hIL-2 and 10 ng/mL hIL-7 were supplemented for another 2 days, and repeat addition cultured for another 3 days. On the 8th day, an equal volume of RPMI-1640 medium was supplemented, and 50 ng/mL hIL-2 was added for further 3 days culture. The induced cells were incubated with pre-loaded with pp65 tumor cells and gradient dilutions of different antibodies for 4 days in 5% CO_2 at 37°C. Stained with PI and killing detection was performed by Flow Cytometry.

In vitro CRS assay

Gradient dilutions of different antibodies, negative control (PBS), and positive control (LPS or a mixture of anti-CD3 and anti-CD28) were added to a 96-well plate according to the experimental design. Subsequently, freshly isolated PBMCs from 3 donors were collected and added at a quantity of 2.5×10^5 cells per well. The plate was then incubated for 24 h in 5% CO_2 at 37°C. The levels of IL-2, IFN-γ, TNF-α, IL-4, IL-6, IL-12p70, and IL-1β in the supernatant were detected using the Legendplex Multiplex Assay Kit (Biolegend) according to the manufacturer's protocol.

Toxicity study in NHPs

For the 4-week repeated-dose toxicity study was conducted in JOINN Laboratories Co., Ltd at GLP compliance. Male, and female, a total of 40 cynomolgus monkeys (20/sex) were treated with anti-PD-1×IL-10M once weekly at dose of 0 (control), 0.5, 2.5 or 10 mg/kg by IV infusion for 5 doses. All available animals (3/sex) were terminated on study Day 30; the rest of animals (2/sex) were necropsied at the completion of 4-week recovery period on day 57. Pharmacokinetic sampling was taken on the indicated days relative to the first dose of anti-PD-1×IL-10M and time from the most recent dose also indicated in hours (h). Plasma cytokine concentrations were determined for IL-18 and IL-6 using an ELISA kit. Body weight, immunophenotyping of peripheral immune populations and cell counts were performed at multiple time point over the course of the study. Hematology analyses including white blood cells (WBCs), red blood cells (RBCs), platelets and reticulocytes (Retic). Peripheral blood safety analyses including alanine transaminase (ALT), aspartate aminotransferase (AST), gamma-glutamyl transferase (GGT), hemoglobin (HGB), and monocyte. Immune cell subset analyses including $CD8^+$ T cells, $CD4^+$ T cells, Treg cells, and NK cells.

QUANTIFICATION AND STATISTICAL ANALYSIS

Quantification and statistical analyses were performed using GraphPad software (Prism 8.3.0, USA). Data are reported as mean ± SEM unless otherwise stated, and significance was assigned at $^*p \leq 0.05$, $^{**}p \leq 0.01$, $^{***}p \leq 0.001$, $^{****}p \leq 0.0001$. The primary statistical analyses performed by two-way ANOVA with Dunnett's multiple comparisons tests, one-way ANOVA with Dunnett's multiple comparisons tests and unpaired Student's t tests. The statistical details of each experiment (including the statistical tests used, sample size *n* values, and statistical significance levels) can be found in the corresponding figures and figure legends.

Cell Reports Medicine, Volume *7*

Supplemental information

An innovative engineered IL-10 monomer strengthens T cell-mediated anti-tumor responses through anti-PD-1 *cis*-delivery

Ce Gu, Jian Guo, Chang Zhou, Peipei Hu, Xiaodong Wu, Jiaojiao Ding, Xinxin Zhou, Liao Zeng, Wen Yu, Yingye Ou, Linhui Ye, Mengying Liang, Yue Huang, Jiatian Li, Zhe Zhang, Wentao Deng, Baiguang Ren, Yingpei Zhang, Li Wang, Xuejiao Chen, Yingxing Duan, Zhe Han, Yang Leng, Hongxin Li, Kongzhen Hu, Yongting Huo, and Di Lu

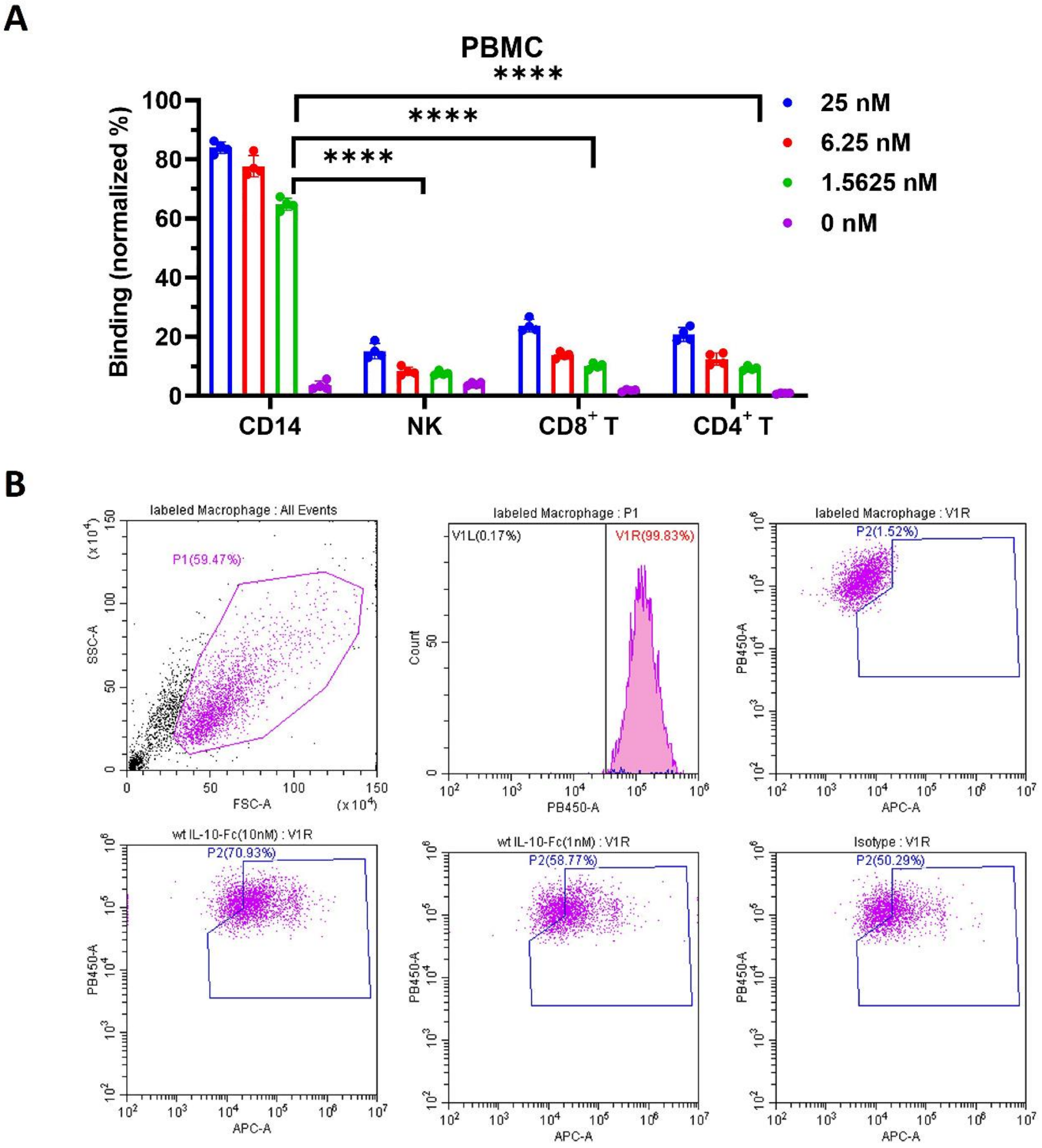

Figure S1. PBMC binding and representative gating strategies for macrophage-mediated RBC uptake. Related to Figure 1.

(A) PBMC cells from another donor were incubated with wt IL-10-Fc in vitro. Protein binding to $CD4^+$ T, $CD8^+$ T, NK or CD14 cells was detected by flow cytometric analysis. (B) Gating was performed on the dual-channel scatter plot. Cells that are $PB450^+$ APC^+ within the P2 gate are macrophages that have phagocytosed red blood cells, and the experiment conducts quantitative and statistical analysis based on the proportion of this cell population. (PB450 is the fluorescence channel corresponding to the macrophage-labeling dye, and APC is the fluorescence channel corresponding to the red blood cell-labeling dye.) Data are reported as the mean ± SEM. Statistical analyses performed by one-way ANOVA with Dunnett's multiple comparisons tests (****$p \leq 0.0001$).

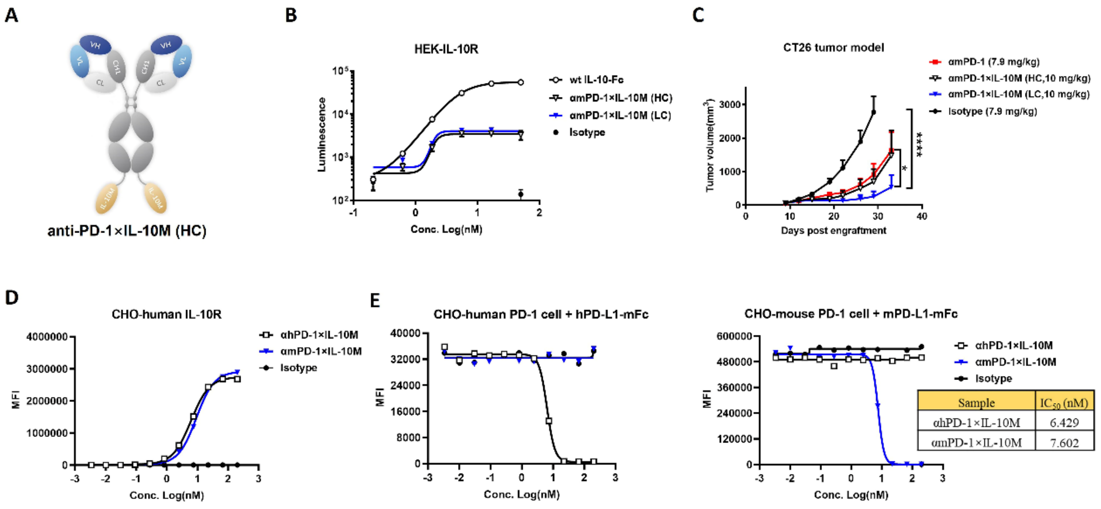

Figure S2. Anti-mPD-1×IL-10M (LC) displayed superior anti-tumor efficacy and exhibited comparable biological activities to anti-hPD-1×IL-10M (LC). Related to Figure 3.

(A) Schematic diagram of anti-PD-1×IL-10M (HC). IL-10M is linked to the C-terminus of the anti-PD-1 antibody heavy chain via (G4S)4G. (B) The bioactivity of the indicated proteins was detected using HEK-IL-10R reporter cells. (C) Balb/c mice were inoculated with 1×10^5 CT26 tumor cells. Tumor-bearing mice (n = 8/group) were intraperitoneally treated with indicated proteins on days 9, 12 and 15. The tumor volume of mice was measured as indicated. (D) Binding activity of indicated proteins to CHO-human IL-10R cells was analyzed by flow cytometric analysis. (E) Blocking activity of indicated proteins on PD-L1 protein binding to CHO-human PD-1 cells (left) or CHO-mouse PD-1 cells (right) was analyzed by flow cytometric analysis. Results are shown as mean ± SEM. Statistical analyses performed by two-way ANOVA with Dunnett's multiple comparisons tests (*$p \leq 0.05$, ****$p \leq 0.0001$).

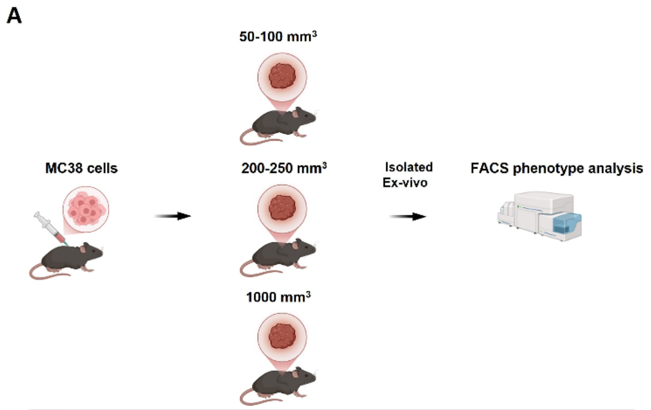

Tumor volume	$CD45^+$ %	$CD8^+$ %	PD-1^+ %	IL-$10R^+$ %	TIM-3^+ %	TOX^+ %
50-100 mm^3	71.83	4.11	52.15	21.63	17.62	34.81
200-250 mm^3	54.52	3.17	64.21	25.04	14.73	39.76
1000 mm^3	46.79	6.04	82.1	46.53	23.86	91.43

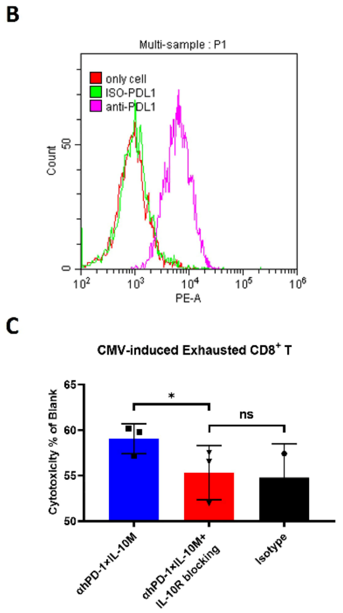

Figure S3. Schematic diagram of isolating tumor infiltrating lymphocytes from MC38 tumors and the expression of PD-L1 and cytotoxicity in PANC.05.04 cells. Related to Figure 4.

(A) C57BL/6 mice were inoculated with 3×10^5 MC38 tumor cells. Tumor infiltrating lymphocytes (TILs) were isolated from MC38 tumor tissues with a volume of 50-100 mm^3, 200-250 mm^3 and 1000 mm^3 using density gradient centrifugation. The proportion of TILs was detected by flow cytometric analysis. Then isolated the $CD8^+$ TILs using mouse CD8 Microbeads (Milton Biotec). The proportion of $CD8^+$, PD-1^+ $CD8^+$, IL-$10R^+$ $CD8^+$, TIM-3^+ $CD8^+$ and TOX^+ $CD8^+$ cells were detected by flow cytometric phenotypic analysis. (B) The expression of PD-L1 in PANC.05.04 cells by flow cytometry. (C) CMV-induced exhausted $CD8^+$ T were co-cultured with PANC pre-loaded with pp65. Flow cytometric analysis was used to detect the killing efficiency of PANC.05.04 tumor cells under the action of the candidate molecule alone and the candidate molecule combined with the IL-10R inhibitor. Results are reported as the mean ± SEM. Statistical analyses performed by paired Student's t test. (ns, not significant, *$p \leq 0.05$).

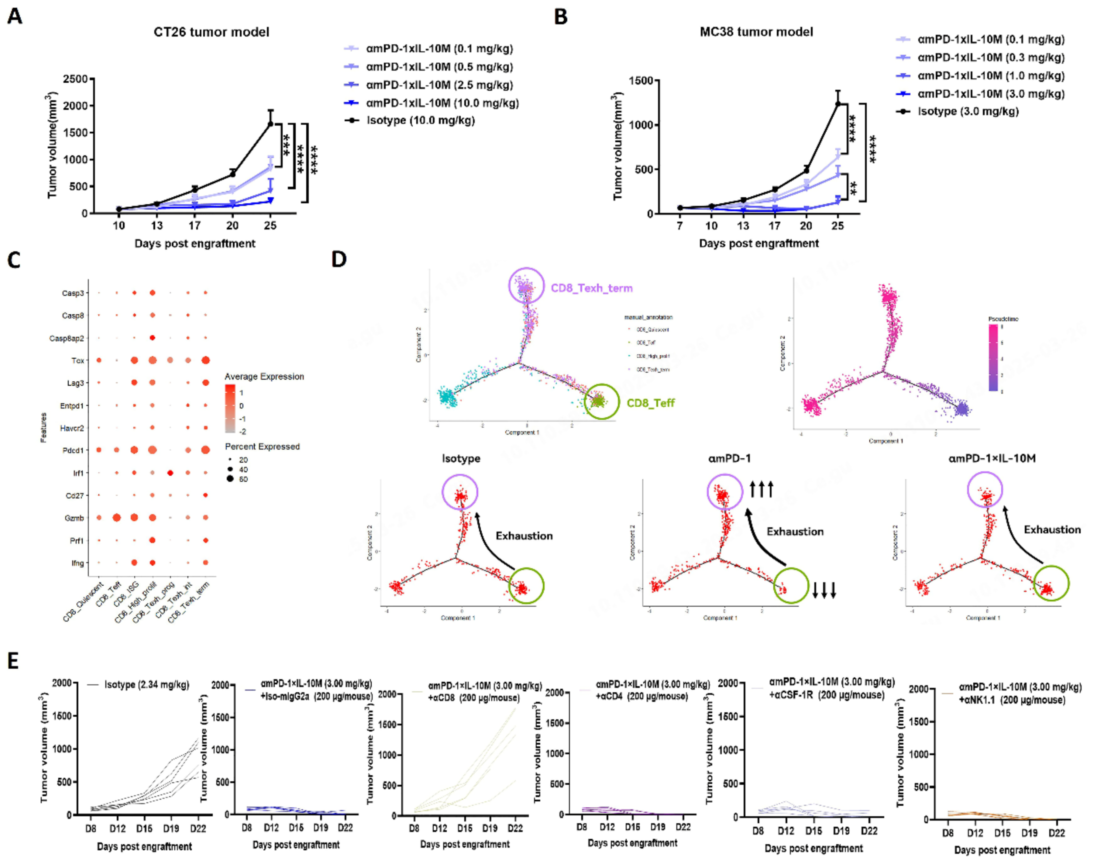

Figure S4. Anti-PD-1×IL-10M exerts anti-tumor efficacy depends on intratumoral CD8$^+$ T cells. Related to Figure 5.

(A) Balb/c mice were inoculated with 1×10^5 CT26 tumor cells. Tumor-bearing mice (n = 9/group) were intraperitoneally treated with different doses of anti-mPD-1×IL-10M proteins on days 10, 13 and 17. The tumor volume of mice was measured as indicated. (B) C57BL/6 mice were inoculated with 3×10^5 MC38 tumor cells. Tumor-bearing mice (n = 9/group) were intraperitoneally treated with different doses of anti-mPD-1×IL-10M proteins on days 7, 10 and 13. The tumor volume of mice was measured as indicated. (C-D) Single-cell sequencing analysis of immune cells in MC38 tumors. (C) According to the expression of corresponding markers, CD8$^+$ T cells were classified into seven types of T cells with different states and functions. CD8_Quiescent, resting CD8$^+$ T cells. CD8_Teff, effector CD8$^+$ T cells. CD8_ISG, expressing interferon responsive genes CD8$^+$ T cells. CD8_High-prolif, high proliferative capacity CD8$^+$ T cells. CD8_Texh-prog, precursor exhausted CD8$^+$ T cells. CD8_Texh-int, intermediate exhausted CD8$^+$ T cells. CD8_Texh-term, terminally exhausted CD8$^+$ T cells. (D) Pseudo temporal analysis of the indicated proteins. (E) Individual tumor growth curves of Fig. 5i. Results are shown as mean ± SEM. Statistical analyses performed by two-way ANOVA with Dunnett's multiple comparisons tests (**$p \leq 0.01$, ***$p \leq 0.001$, ****$p \leq 0.0001$).

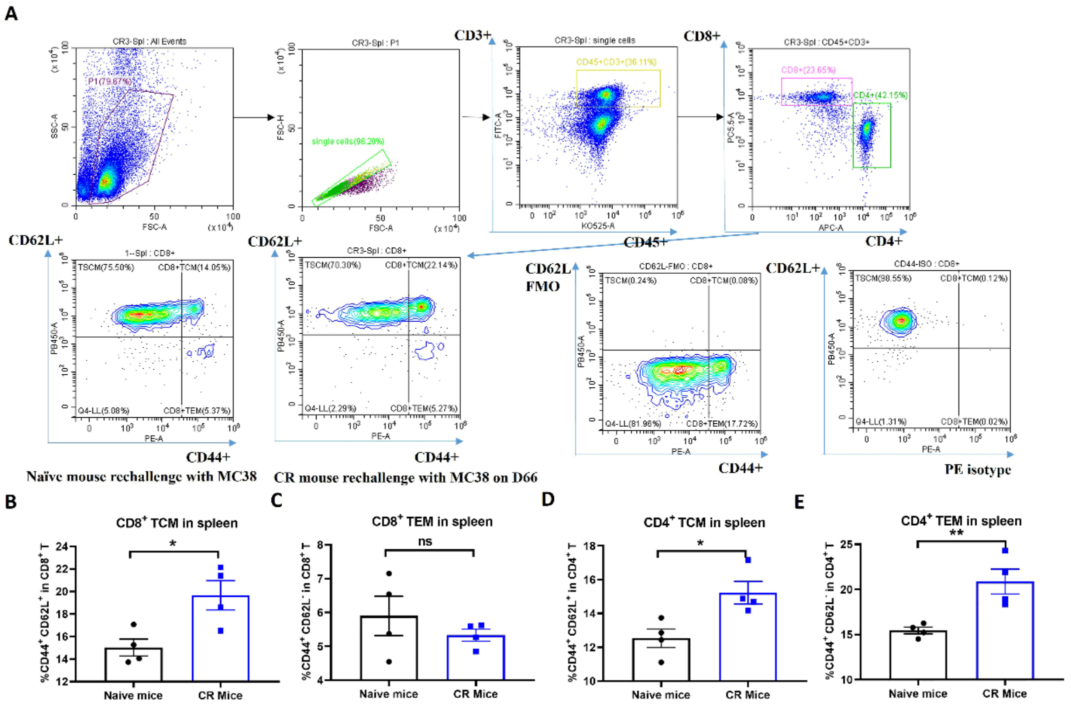

Figure S5. Representative gating strategies and flow cytometry analysis for immunophenotyping of murine spleen samples. Related to Figure 6.

Splenocytes were isolated from treated mice and the immunophenotyping of cell populations was conducted. (A) Gating of $CD8^+$ T and $CD4^+$ T cell populations, namely central memory $CD8^+$ T cells ($CD8^+$ TCM; $CD3^+/CD8^+/CD44^+/CD62L^+$), central memory $CD4^+$ T cells ($CD4^+$ TCM; $CD3^+/CD4^+/CD44^+/CD62L^+$), effector memory $CD8^+$ T cells ($CD8^+$ TEM; $CD3^+/CD8^+/CD44^+/CD62L^-$) and effector memory $CD4^+$ T cells ($CD4^+$ TEM; $CD3^+/CD4^+/CD44^+/CD62L^-$). (B-E) After rechallenging the cured mice with MC38 tumor cells, the changes in the proportion of memory T cells in the mice's spleens were systematically analyzed using flow cytometry. Results are shown as mean ± SEM. Statistical analyses performed by one-way ANOVA with Dunnett's multiple comparisons tests (ns, not significant; $*p \leq 0.05$; $**p \leq 0.01$). CR (Complete Response); Spl (Splenocytes).

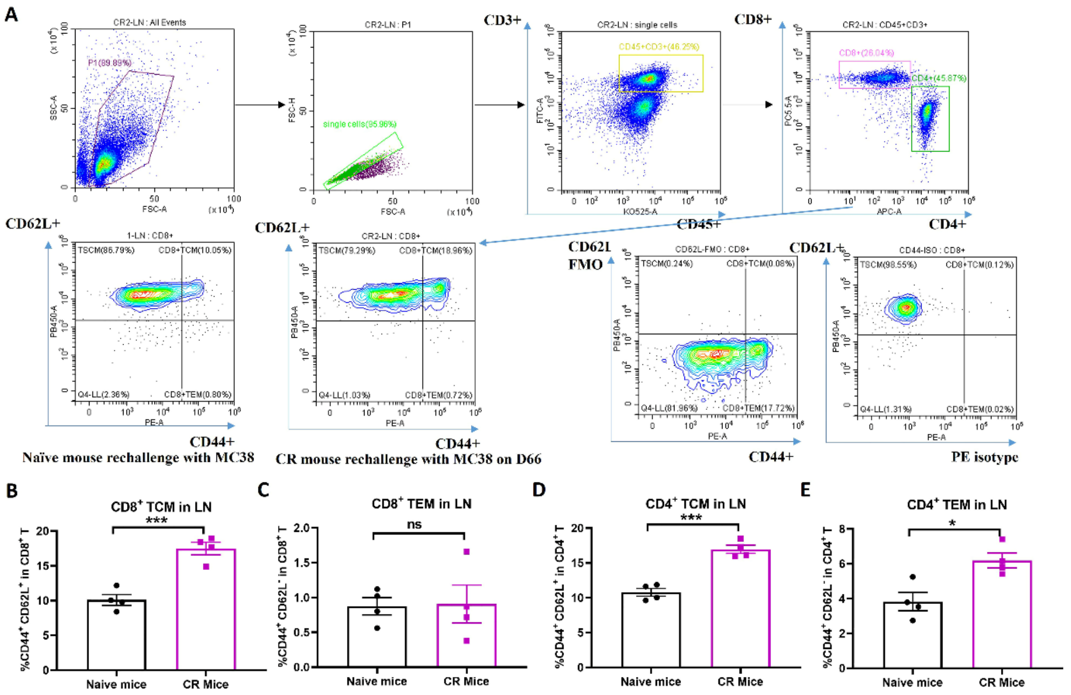

Figure S6. Representative gating strategies and flow cytometry analysis for immunophenotyping of murine lymph node samples. Related to Figure 6.

Lymph node cells were isolated from treated mice and the immunophenotyping of cell populations was conducted. (A) Gating of $CD8^+$ T and $CD4^+$ T cell populations, namely central memory $CD8^+$ T cells ($CD8^+$ TCM; $CD3^+/CD8^+/CD44^+/CD62L^+$), central memory $CD4^+$ T cells ($CD4^+$ TCM; $CD3^+/CD4^+/CD44^+/CD62L^+$), effector memory $CD8^+$ T cells ($CD8^+$ TEM; $CD3^+/CD8^+/CD44^+/CD62L^-$) and effector memory $CD4^+$ T cells ($CD4^+$ TEM; $CD3^+/CD4^+/CD44^+/CD62L^-$). (B-E) After rechallenging the cured mice with MC38 tumor cells, the changes in the proportion of memory T cells in the mice's lymph nodes were systematically analyzed using flow cytometry. Results are shown as mean ± SEM. Statistical analyses performed by one-way ANOVA with Dunnett's multiple comparisons tests (ns, not significant; $*p \leq 0.05$; $***p \leq 0.001$). CR (Complete Response); LN (Lymph node).

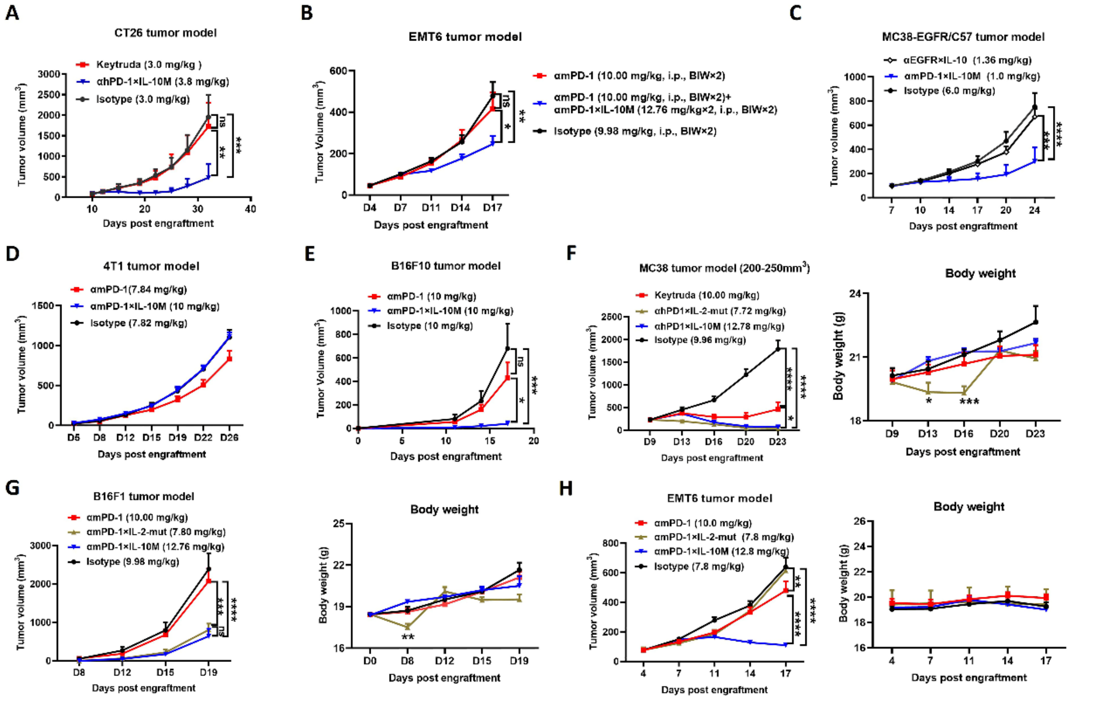

Figure S7. Anti-tumor efficacy of anti-PD-1×IL-10M in various tumor models. Related to Figure 6.

(A) Balb/c-hPD-1 mice were inoculated with 1×10^5 CT26 tumor cells. Tumor-bearing mice (n = 8/group) were intraperitoneally treated with indicated proteins on days 10, 13, 16 and 19. The tumor volume of mice was measured as indicated. (B) Balb/c mice were inoculated with 1×10^6 EMT6 tumor cells. Tumor-bearing mice (n = 8/group) were intraperitoneally treated with anti-mPD-1 proteins on days 4 and 7, anti-mPD-1×IL-10M proteins on days 11and 14. (C) C57BL/6 mice were inoculated with 1×10^6 MC38-EGFR tumor cells. Tumor-bearing mice (n = 8/group) were intraperitoneally treated with indicated proteins on days 7, 10, 14 and 17. (D) Balb/c mice were inoculated with 2×10^5 4T1 tumor cells. Tumor-bearing mice (n = 6/group) were intraperitoneally treated with indicated proteins on days 5, 8 and 12. (E) C57BL/6 mice were inoculated with 2×10^5 B16F10 tumor cells. Tumor-bearing mice (n = 7/group) were intraperitoneally treated with indicated proteins on days 0, 2 and 4. (F) C57BL/6-hPD1 mice were inoculated with 1×10^6 MC38 tumor cells. Tumor-bearing mice (n = 8/group) were intraperitoneally treated with indicated proteins on days 9, 13 and 16. The tumor volume and body weight of mice was measured as indicated. (G) C57BL/6 mice were inoculated with 2×10^5 B16F1 tumor cells. Tumor-bearing mice (n = 8/group) were intraperitoneally treated with indicated proteins on days 0, 2 and 4. The tumor volume and body weight of mice was measured as indicated. (H) Balb/c mice were inoculated with 1×10^6 EMT6 tumor cells. Tumor-bearing mice (n = 9/group) were intraperitoneally treated with indicated proteins on days 4, 7 and 11. The tumor volume and body weight of mice was measured as indicated. Results are shown as mean ± SEM. Statistical analyses performed by two-way ANOVA with Dunnett's multiple comparisons tests (ns, not significant, *$p \leq 0.05$, **$p \leq 0.01$, ***$p \leq 0.001$, ****$p \leq 0.0001$).
